# Tissue-resident memory T cells in epicardial adipose tissue comprise transcriptionally distinct subsets that are modulated in atrial fibrillation

**DOI:** 10.1038/s44161-024-00532-x

**Published:** 2024-08-23

**Authors:** Vishal Vyas, Balraj Sandhar, Jack M. Keane, Elizabeth G. Wood, Hazel Blythe, Aled Jones, Eriomina Shahaj, Silvia Fanti, Jack Williams, Nasrine Metic, Mirjana Efremova, Han Leng Ng, Gayathri Nageswaran, Suzanne Byrne, Niklas Feldhahn, Federica Marelli-Berg, Benny Chain, Andrew Tinker, Malcolm C. Finlay, M. Paula Longhi

**Affiliations:** 1 William Harvey Research Institute, Barts and The London School of Medicine and Dentistry, Queen Mary University of London, London, UK; 2 Department of Cardiology, Barts Heart Centre, St. Bartholomew’s Hospital, London, UK; 3 Cancer Research UK, Barts Centre, Queen Mary University of London, London, UK; 4 Department of Immunology and Inflammation, Centre for Haematology, Faculty of Medicine, Imperial College London, London, UK; 5 UCL Division of Infection and Immunity, University College London, London, UK

**Keywords:** Atrial fibrillation, Inflammation, Lymphocyte activation

## Abstract

Atrial fibrillation (AF) is the most common sustained arrhythmia and carries an increased risk of stroke and heart failure. Here we investigated how the immune infiltrate of human epicardial adipose tissue (EAT), which directly overlies the myocardium, contributes to AF. Flow cytometry analysis revealed an enrichment of tissue-resident memory T (T_RM_) cells in patients with AF. Cellular indexing of transcriptomes and epitopes by sequencing (CITE-seq) and single-cell T cell receptor (TCR) sequencing identified two transcriptionally distinct CD8^+^ T_RM_ cells that are modulated in AF. Spatial transcriptomic analysis of EAT and atrial tissue identified the border region between the tissues to be a region of intense inflammatory and fibrotic activity, and the addition of T_RM_ populations to atrial cardiomyocytes demonstrated their ability to differentially alter calcium flux as well as activate inflammatory and apoptotic signaling pathways. This study identified EAT as a reservoir of T_RM_ cells that can directly modulate vulnerability to cardiac arrhythmia.

## Abstract (web summary)

Vyas et al. show that epicardial adipose tissue is a reservoir for a subpopulation of tissue-resident memory T cells that can increase the vulnerability of the heart to atrial fibrillation.

## Main

Atrial fibrillation (AF) is the most common sustained arrhythmia worldwide, defined by rapid, uncoordinated atrial activity with consequent deterioration of atrial mechanical function^1^. Patients suffering from AF have poorer outcomes in heart failure, an increased risk of cognitive decline and vascular dementia as well as a five-fold increased risk of stroke^2,3^. The financial costs are commensurate with this health burden, with over £2 billion spent annually in healthcare costs within England alone^4^.

The exact etiology of AF remains incompletely understood, requiring complex interactions between triggers and the underlying atrial substrate to sustain AF. Inflammation is known to play a key role in the formation of AF substrate, promoting electrical and structural remodeling of the atrium and increasing vulnerability to AF^5^. Several studies described an association between AF and serum inflammatory biomarkers, such as C-reactive protein (CRP) and IL-6 (refs. ^6,7^). However, only a limited number of studies have looked at the immune infiltrate in the atrial tissue itself. Abnormal atrial histology, characterized by inflammatory infiltrates, fibrosis and expression of pro-inflammatory cytokines, has been identified in tissue biopsies of patients with AF^8–11^. However, the patient numbers and range of tissue analyses in these studies remain limited.

Numerous lines of evidence suggest a role of epicardial adipose tissue (EAT) in the development of AF. EAT is the visceral fat depot of the heart that shares direct anatomic contact with the myocardium without fascial interruption. EAT has been demonstrated to be a significant source of inflammatory mediators that can exert paracrine and vasocrine effects on the myocardium^12^. Several observational studies have demonstrated that EAT volume is consistently associated with the presence, severity and recurrence of AF^13–15^. Furthermore, increased EAT inflammation, as measured by ^18^F-fluorodeoxyglucose (FDG) uptake, was observed in patients with AF compared to those in sinus rhythm (SR)^16,17^. A range of pathophysiological mechanisms could contribute to the association between EAT and AF, including adipocyte infiltration, oxidative stress and the paracrine effect of pro-fibrotic and pro-inflammatory cytokines. However, the exact immune structure and cellular characterization of EAT in AF remains elusive.

In the present study, we investigated the pathophysiological significance of the immune infiltrate in the EAT of patients with AF. Flow cytometry analysis identified an enrichment of tissue-resident memory T (T_RM_) cells in patients with AF compared to SR controls. T_RM_ cells are a specialized subset of memory T cells that persist long term in peripheral tissues with minimal recirculation. To further characterize these cells, we applied cellular indexing of transcriptomes and epitopes by sequencing (CITE-seq) combined with single-cell T cell receptor (TCR) sequencing, which identified two transcriptionally distinct CD8^+^ T_RM_ cells that are modulated in AF. Furthermore, spatial transcriptomic analysis of the border zone between the EAT and the atrial, together with functional analysis, suggests that EAT is a reservoir of T_RM_ cells that can serve as mediators of inflammation in the myocardium.

## Results

### T_RM_ cells are increased in the EAT of patients with AF

**Fig. 1:**
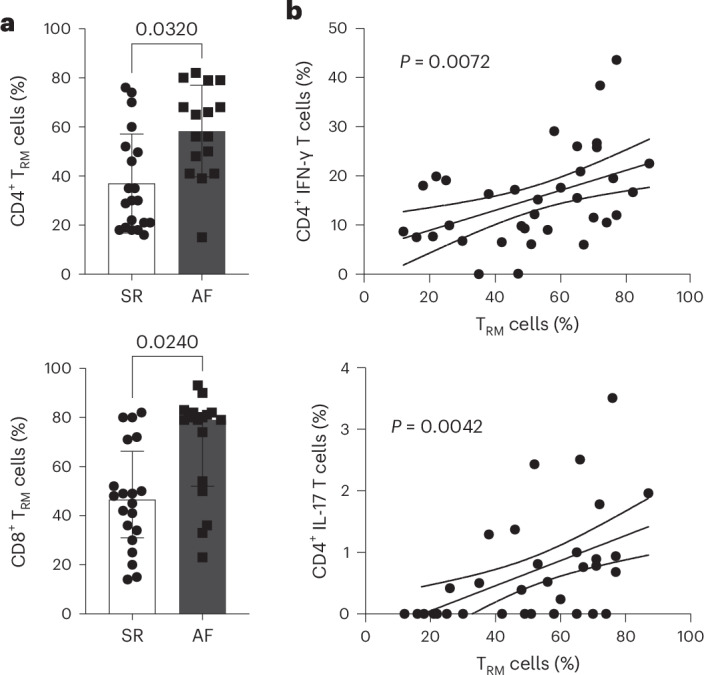
Evaluation of T_RM_ cell frequency in EAT. EAT immune infiltrate from patients with AF or patients in SR was characterized by flow cytometry. **a**, Bar graphs indicate the frequency of CD4^+^ and CD8^+^ T_RM_ cells over total CD4^+^ or CD8^+^CD45RO^+^ T cells, respectively, in the EAT (*n* = 26 SR and *n* = 18 AF biological replicates). Statistical significance was determined using unpaired two-tailed *t*-test for the parametrically distributed groups. Data are represented as mean ± s.d. **b**, Cytokine production was evaluated by intracellular staining by flow cytometry. Correlation analysis of IFN-γ and IL-17 production with the frequency of CD4^+^ T_RM_ cells measured by linear regression. Graphs show 95% confidence bands (*r* = 0.299) and two-tailed *P* value analysis. All data show individual patients (*n* = 44).

**Extended Data Fig. 1:**
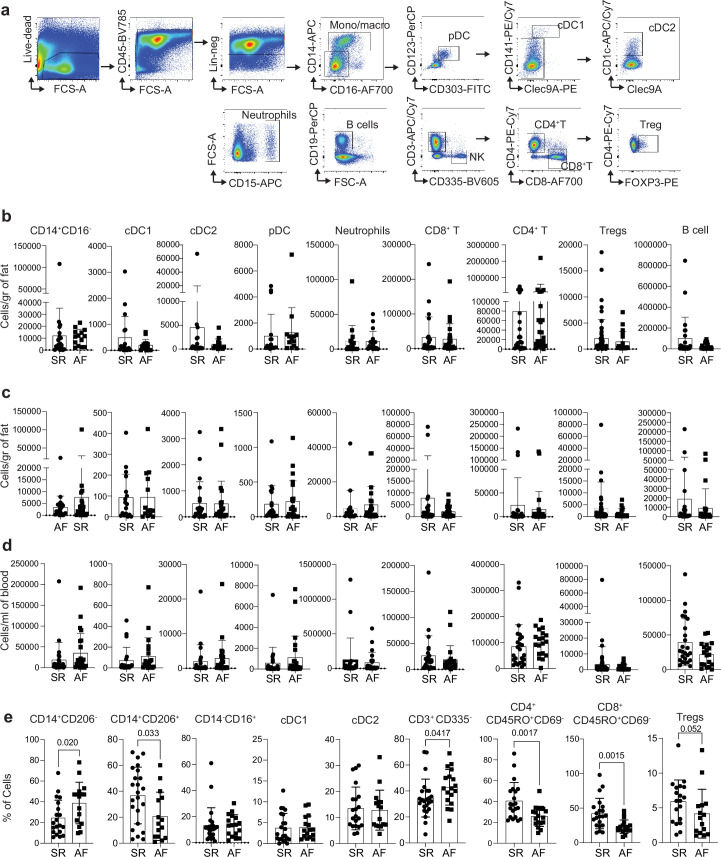
Immune profiling of EAT. **a**. Gating strategy for the identification of lymphoid and myeloid cells in tissue. **b-d**. Absolute number of immune cells in patients with AF and on SR across EAT (**b**), SAT (**c**) and blood (**d**). **e**. Percentages of myeloid and lymphoid cells in the EAT. Statistical significance was determined by the unpaired two-tailed Mann-Whitney test, and data are represented as Mean ± SD. **b-e**. Data shows individual patients (n=26 SN and 18 AF biological replicates).

**Extended Data Fig. 2.**
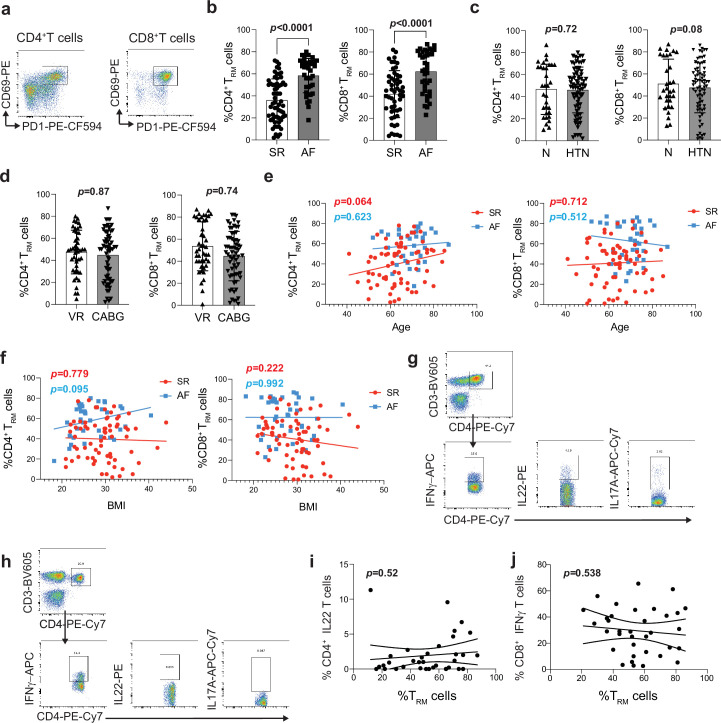
Frequency of T_RM_ cells in AF. **a**. Gating strategy for the identification of T_RM_ cells. Immune cells were gated on live CD3^+^CD4^+^ or CD8^+^ T cells as shown in Extended Data Fig. 1a. **b**. Frequency of CD4^+^ and CD8^+^ T_RM_ cells in unmatched patients with AF compared to SR controls. Patients with post-operative AF were excluded. **c**. Frequency of CD4^+^ and CD8^+^ T_RM_ cells in normotensive (N) compared to hypertensive (HTN) patients. **d**. Frequency of CD4^+^ and CD8^+^ T_RM_ cells between patients undergoing VR or CABG surgery. **b-d**. Significance was obtained using two-tailed Mann-Whitney test. Data is represented as Mean ± SD **e-f**. Correlation analysis of CD4^+^ and CD8^+^ T_RM_ cells with age (**d**) and BMI (**e)** by lineal regression and two-tailed p-value analysis**. g-h**. Gating strategy for the identification of cytokine production by CD4^+^ (**f**) or CD8^+^ (**g**) T cells T_RM_ cells. **i**. Correlation analysis of IL22 production with the frequency of CD4^+^ T_RM_ cells measured by lineal regression and two-tailed p-value analysis. **j**. Correlation analysis of IFNγ production with the frequency of CD8^+^ T_RM_ cells measured by lineal regression and two-tailed p-value analysis. **b-i**. Data shows individual patients (n=122).

A total of 153 participants undergoing open heart surgery were recruited to this study. The mean age was 66.1 years, with a mean body mass index (BMI) of 28.2 kg m^−2^, and 75% of participants were male, with 50% undergoing a coronary artery bypass surgery (Supplementary Table 1). To evaluate the immune profile of the EAT in AF, participants were classified into two groups based on their 12-lead electrocardiogram (ECG)-confirmed rhythm, recorded preoperatively: AF or those in normal rhythm (SR). Thirty-one participants developed postoperative AF and were, therefore, excluded from the study. Patients with AF were older and more likely to undergo valve surgery (Supplementary Table 2). As expected, patients with AF had a more dilated left atrium, as previously described, as a result of tissue remodeling^18^. Thus, to account for key variables, we performed propensity score matching with age, gender, BMI, diabetes, hypertension and procedure type as covariates (Table 1). Key immune cells were identified by flow cytometry in the EAT, in subcutaneous adipose tissue (SAT) as a control adipose tissue and in blood as a marker of the systemic inflammatory state. No differences were observed in cell number between the groups and across the tissues (Extended Data Fig. 1a–d). The frequencies of myeloid and lymphoid cells were largely unchanged with the exception of a decrease in CD14^+^CD206^+^ macrophages and an increase in total CD3^+^ T cells in patients with AF (Extended Data Fig. 1e). We previously showed that T cells are a predominant immune cell constituent in human EAT^19^. Analysis of T cell subsets identified an increase in T_RM_ cells, as defined by high expression of CD69 and the inhibitory molecule programmed cell death 1 (PD1) receptor, in the EAT of patients with AF compared to SR (Fig. 1a and Extended Data Fig. 2a), which was accompanied by a decrease in CD69^−^ memory T cells (Extended Data Fig. 1e). The increase in T_RM_ cells was readily evident in unmatched participants and independent of risk factors, such as age, hypertension and body weight (Extended Data Fig. 2b–e). T_RM_ cells are transcriptionally programmed for strong effector function to confer swift on-site immune protection^20^. We then evaluated T cell cytokine production by intracellular staining. The gating strategy is shown in Extended Data Fig. 2f,g. A clear correlation between IFN-γ and IL-17 production and the presence of CD4^+^ T_RM_ cells was observed (Fig. 1b). No such correlation was detected for IFN-γ and CD8^+^ T_RM_ cells, which could be attributed to the high cytotoxic activity of these cells (Extended Data Fig. 2h,i). Overall, our data suggest that T_RM_ cells could play a pathological role in the development and/or persistence of AF.

**Table 1:** Clinical characteristic of patients with AF and on SR

	Pre-existing AF (*n* = 18)	SR (*n* = 26)	*P* value
Age (years)	67.3 ± 7.8	65.6 ± 8.8	0.47
Male gender (%)	14 (78)	17 (65)	0.51
BMI (kg m^−2^)	26.6 ± 4.5	26.8 ± 3.9	0.91
Diabetes (%)	2 (11)	3 (12)	0.99
Hypertension (%)	12 (67)	18 (67)	0.99
Hyperlipidaemia (%)	11 (61)	16 (61)	0.99
Smoking history (%)	8 (44)	13 (50)	0.59
Prior myocardial infarction (%)	3 (17)	7 (27)	0.49
Left ventricular ejection fraction (%)	59 (55–63)	60 (58–61)	0.49
Preoperative use of beta-blockers (%)	11 (61)	12 (46)	0.37
Preoperative use of statins (%)	12 (67)	17 (65)	0.99
Preoperative CRP (mg l^−1^)	0 (0–5)	0 (0–9)	0.69
Preoperative neutrophil:lymphocyte	2.6 (1.8–4.2)	2.6 (1.9–3.4)	0.62
Left atrial size (cm)	4.9 ± 1.5	3.6 ± 0.6	0.03*
Coronary artery bypass surgery (%)	9 (50)	50 (67)	0.99
Valve surgery (%)	10 (56)	15 (58)	0.99
Combination coronary artery bypass/valve surgery (%)	3 (17)	3 (12)	0.68

Two-tailed Student’s *t*-test/Mann–Whitney test for continuous data or χ^2^/Fisher’s exact test for categorical data was used where appropriate. *P < 0.05.

### EAT serves as a reservoir of T_RM_ cells

**Fig. 2:**
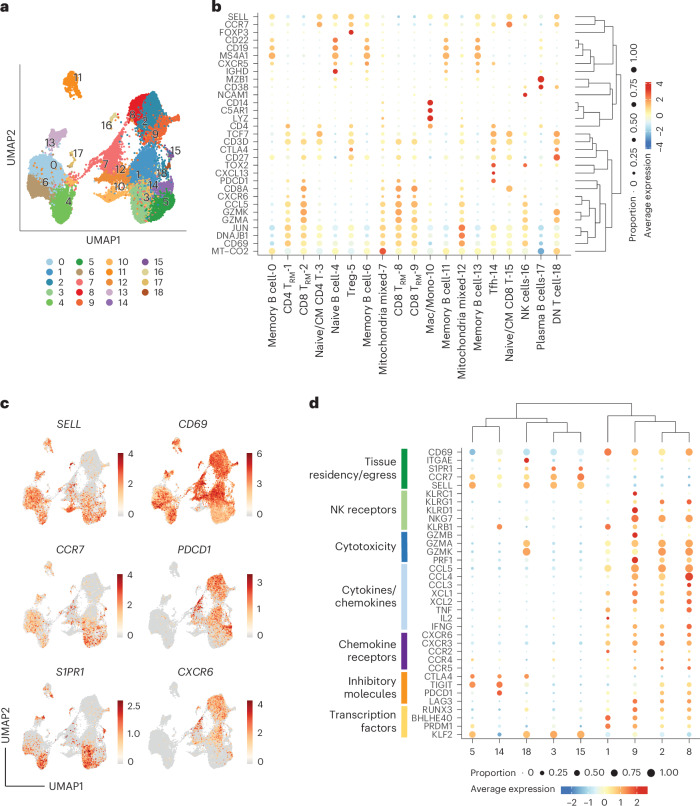
CITE-seq analysis of immune cells in the EAT and AA. **a**, UMAP plots of merged EAT and AA samples identified 19 cell clusters. **b**, Bubble plot shows expression levels of representative markers within each cluster. **c**, Canonical markers used to identify T_RM_ cells are represented in the UMAP plot. Data are colored according to average expression levels. Expression values are normalized for quantitative comparison within each dataset. **d**, Bubble plot showing the expression distribution of effector molecules, receptors and transcription factors among T cell populations. DN, double-negative.

**Extended Data Fig. 3:**
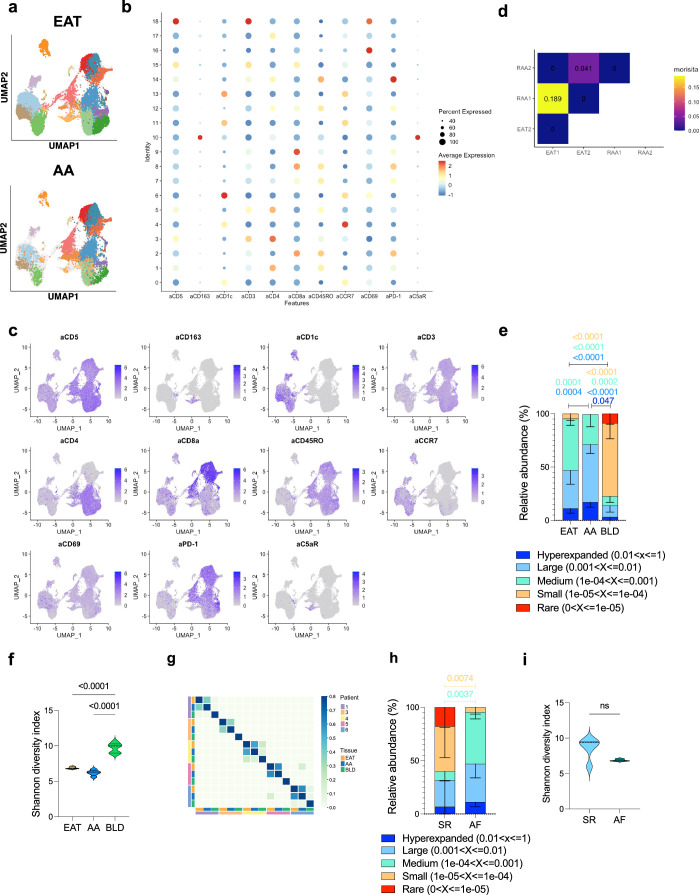
Distinct protein profile across immune cells in EAT and AA. **a**. UMAP plots of EAT and AA samples identifying similar 19 cell clusters. **b**. Bubbleplotshowing surface expression of antibody-derived tag protein expression value for selective markers. **c**. UMAP plot representation of antibody-derived tag protein expression value for selective markers for the identification of memory T cells, B and myeloid cells. Colour bar key represents average expression levels. Expression values are normalized for quantitative comparison within each dataset. **d**. TCR clonal overlap detected by the Morisita index. **e**. Bar graph indicates the relative abundance of TCRβ clonotypes in paired tissues EAT, AA and Blood (BLD). The relative abundance of TCRβ clonotypes. **f**. TCRβ diversity between paired tissues. Statistical significance was evaluated with 2-way Anova followed Benjamini-Hochberg-correction. **g**. Heatmap illustrating the compositional TCRβ similarity between paired samples assessed using the Morisita-Horn index. Colour bar key represents the Morisita index. **h**. Bar graph indicates the relative abundance of TCRβ clonotypes between patients with AF and on SR. Data are presented as mean values ± SD. **e-h**. Statistical significance was evaluated using 2-way ANOVA with Sidak’s multiple comparisons test. **i**. TCRβ diversity between AF and SR patients. Statistical significance was evaluated using the two-tailed Mann-Whitney *U* test for nonparametric data and represented as mean± SD. **e-i**. Data are from 5 biological replicates.

T_RM_ cells are transcriptionally, phenotypically and functionally distinct from other memory T cell populations. T_RM_ cells can be identified in tissue by their expression of CD69 and a core gene signature shared between CD4^+^ and CD8^+^ T_RM_ cells in multiple lymphoid and mucosal sites^20^. However, to establish long-term residency in different tissues, T_RM_ cells are required to display tissue-specific transcriptional features to accommodate unique local environmental cues^21^. To confirm the identity of CD69^+^PD1^+^ cells identified by flow cytometry, we performed CITE-seq profiling of immune cells within the EAT from two patients with AF. CITE-seq allows optimal annotation of cell populations and identification of protein isoforms, such as the canonical memory marker CD45RO, that cannot be identified by RNA sequencing (RNA-seq) alone^22^. In addition, to explore the interrelationship between T cells in the EAT and underlying myocardium, given the anatomical intimacy between these two tissues, we transcriptionally profiled paired EAT and atrial appendage (AA) samples. To improve sensitivity, given that immune cells comprise a relatively small proportion of cells in the EAT and AA, CITE-seq was performed on sorted CD45^+^ cells. Unsupervised clustering and uniform manifold approximation and projection (UMAP) dimensionality reduction from 28,242 cells across the two paired EAT and RAA samples yielded 19 clusters that were annotated based on the expression of data-driven marker genes (Fig. 2a). Similar clusters could be observed in the EAT and paired AA, with a clear enrichment of adaptive immune cells, consistent with our previous work (Extended Data Fig. 3a)^19^.

Differential gene expression from the CITE-seq data resolved T cell subsets into nine clusters (Fig. 2a,b and Supplementary Table 3). CD4^+^ T cells comprised four clusters: a distinct T_RM_ cluster expressing the canonical markers *CD69*, *PDCD1* and *CXCR6* and low *CCR7* and *SELL* expression (cluster 1); naive or T central memory (T_CM_) cells expressing *CCR7*, *SELL* and *TCF7* (cluster 3); a cluster representing regulatory T cells (Tregs) expressing *FOXP3* and *CTLA4* (cluster 5); and a T follicular helper cell (Tfh) cluster characterized by the expression of *CXCL13*, *TOX2*, *CXCR5* and *PDCD1* (cluster 14). CD8^+^ T cells comprised four clusters that included the following: three T_RM_ clusters expressing *CCL5*, the T_RM_ markers *CD69*, *PDCD1* and *CXCR6* and the cytotoxic-associated genes *GZMK* and *GZMA* (clusters 2, 8 and 9); and naive or T_CM_ cells expressing *CCR7*, *SELL* and *TCF7* (cluster 15). A small double-negative T cell cluster was identified by the expression of *CD3D* and absence of *CD4* and *CD8A* expression (cluster 18). In addition, we identified five B cell clusters based on the expression of *CD19*, *CD22* and *MS4A1*, with memory B cells expressing *CD27* and naive B cells expressing *IGHD*; high expression of *CD38* and *MZB1* defined a cluster of plasma B cells. A single cluster of monocytes/macrophages was identified based on the expression of *C5AR1*, *LYZ* and *CD14*, and a natural killer (NK) cell cluster was defined by the expression of *NCAM1*. Two clusters were found to be enriched in mitochondrial and heat shock protein genes indicative of a stress-like state and were integrated by a mixed population of monocytes and T cells. Expression of surface markers detected by TotalSeq antibodies (oligonucleotide-tagged antibodies) confirmed the expression of CD45RO on memory T cells as well as PD1 and CD69 expression on T_RM_ cells, which were also low on CCR7 (Extended Data Fig. 3b,c).

Consistent with our flow cytometry data, T_RM_ cells made up a sizeable proportion of the T cell repertoire, in particular for CD8^+^ T cell populations. As expected, they differentially express genes associated with tissue retention/egress, but they lack expression of CD103, which is normally expressed on CD8^+^ T_RM_ cells at the epithelial barrier^23^ (Fig. 2c,d). T_RM_ cells exhibit constitutively high expression of deployment-ready mRNAs encoding effector molecules, such as granzymes, cytokines and chemokines, enabling rapid immune responses (Fig. 2d). Thus, to control their undue activation, they express the inhibitory molecules *LAG3* and *PDCD1* but lack the expression of *CTLA4* (ref. ^20^). Thus, CITE-seq provided the necessary single-cell resolution to demonstrate a gene signature consistent with that observed in other organs, such as the lung, gut and skin^24^, and demonstrated that the elevated CD69^+^PD1^+^ T cell population observed in patients with AF is consistent with a T_RM_ cell phenotype with high effector cytotoxic function.

### T_RM_ cells are recruited into the atrial myocardium

**Fig. 3:**
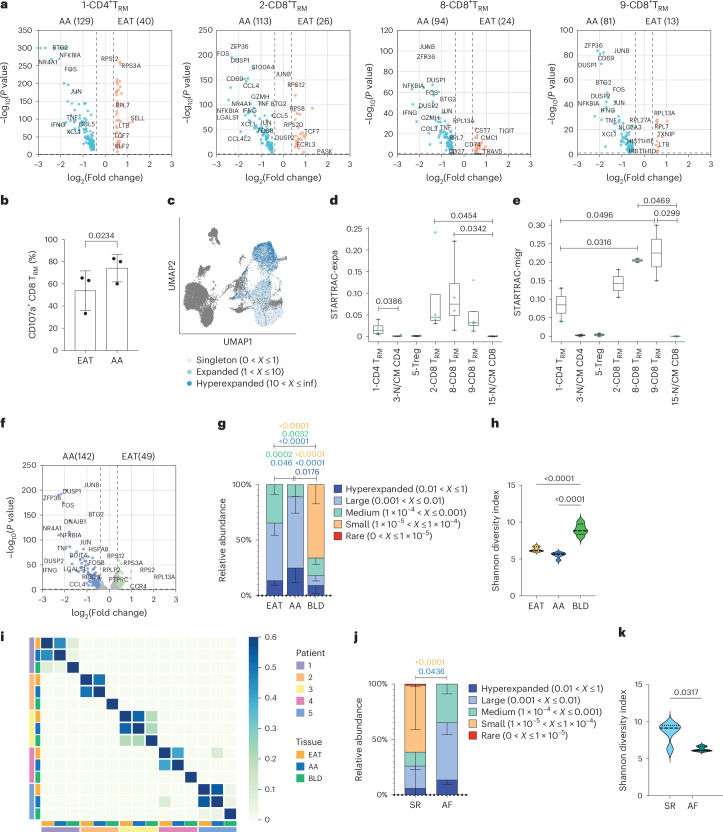
Characterization of T_RM_ cells across EAT and AA tissues. **a**, Volcano plots showing the average log fold changes and average Benjamini–Hochberg-corrected *P* values for pairwise differential expression between EAT and AA tissues for all T_RM_ cluster populations based on the non-parametric Wilcoxon rank-sum test. **b**, Expression of surface CD107a was analyzed on activated CD8^+^ T_RM_ cells by flow cytometry. Bar graph indicates the percentage of CD107a^+^CD8^+^ T_RM_ cells in paired EAT and AA samples. Data are presented as mean ± s.d. Statistical significance was determined using paired two-tailed *t*-test (*n* = 3 biological replicates). **c**, UMAP visualization of clonotype expansion levels among clusters. Data are colored according to clonal expansion levels. **d**, Clonal expansion levels of T cell clusters quantified by STARTRAC-expa indices for each sample. Statistical significance was determined using the Kruskal–Wallis test with Dunn’s multiple comparisons test (*n* = 4 biological replicates) **e**, Migration potential of T cell clusters quantified by STARTRAC-migr indices for each patient. Statistical significance was determined using one-way ANOVA with Tukey’s multiple comparisons test (*n* = 2 biological replicates). Box plots in **d** and **e** show data points from individual tissues with means and minimum/maximum values. **f**, Volcano plots showing the average log fold changes and average Benjamini–Hochberg-corrected *P* values for pairwise differential expression between hyperexpanded TCR clones in the EAT and AA tissues. **g**, Bar graph indicates the relative abundance of TCRα clonotypes in paired tissues EAT, AA and blood (BLD) (*n* = 5 biological replicates). The relative abundance of TCRα clonotypes was calculated using the Immunarch package in R (version 1.0.0) and grouped accordingly as rare, small, medium, large and hyperexpanded. Data are presented as mean ± s.d. Statistical significance was evaluated using two-way ANOVA with Sidak’s multiple comparisons test. **h**, TCRα diversity between paired tissues (*n* = 5 biological replicates). Statistical significance was evaluated with one-way ANOVA followed by the Tukey’s multiple comparison test. **i**, Heatmap illustrating the compositional TCRα similarity between paired samples assessed using the Morisita–Horn index. **j**, Bar graph indicates the relative abundance of TCRα clonotypes between patients with AF and patients in SR (*n* = 5 biological replicates). Data are presented as mean values ± s.d. Statistical significance was evaluated using two-way ANOVA with Sidak’s multiple comparisons test. **k**, TCRα diversity between patients with AF and patients in SR (*n* = 5 biological replicates). Statistical significance was evaluated using the two-tailed Mann–Whitney *U*-test for non-parametric data and represented as mean ± s.d. Panels **h**–**k** show medians, and light dotted lines show 1st and 3rd quartiles. inf, infinity. Source data

The EAT is now considered an immune site harboring an array of innate and adaptive immune cells and is thought to act as a reservoir for memory T cells^25^. Due to the absence of fascial boundaries and close functional and anatomic relationship, T cells present in the EAT could migrate and exert a detrimental effect on the myocardium. To characterize the T_RM_ cell populations between tissues, we identified differentially expressed genes (DEGs) for all T_RM_ cell clusters (Supplementary Table 4). Supporting the strong immune crosstalk between tissues, T_RM_ cells in EAT and AA showed a similar core phenotype. However, DEG analysis revealed an upregulation of activated genes—for example, *JUNB*, *FOS*, *ZFP36* and *IFNG*—in the T_RM_ cells present in the AA (Fig. 3a)^26^, which could be confirmed by increased degranulation of CD8^+^ T_RM_ cells (Fig. 3b). This activated phenotype was not restricted to T_RM_ cells but observed across immune cell clusters (Supplementary Table 4).

To further evaluate the dynamic relationship of T cells among EAT and AA tissues, CITE-seq was combined with TCR α-chain and β-chain sequencing. Distinct clonotypes were assigned according to the presence of unique nucleotide sequences for both α and β chains. A distinct pattern of T cell clonal expansion could be observed, with T_RM_ cells showing the highest degree of clonal expansion, in particular CD8^+^ T_RM_ cells (Fig. 3c,d and Supplementary Table 5). STARTRAC-migr analysis revealed that T_RM_ cells are associated with the highest mobility with a high degree of TCR sharing between EAT and AA (Fig. 3e)^27^. TCR similarity was confirmed with the Morisita index (Extended Data Fig. 3d). To understand the trajectory of these cells, we performed DEG analysis on shared expanded clones between EAT and AA. Shared expanded TCR clones in the AA upregulate expression of *JUNB*, *FOS*, *IFNG*, *TNF* and *ZFP36* compared to EAT, which is consistent with T cell activation (Fig. 3f and Supplementary Table 6).

To confirm these findings, bulk TCRα-β sequencing was performed on matched blood, EAT and AA samples from six participants. Greater clonal expansion and lower clonotype diversity were detected in EAT and AA samples compared to blood (Fig. 3g,h and Extended Data Fig. 3e,f). In addition, a high degree of TCR similarity could be detected between EAT and AA paired samples, whereas a relative low proportion of shared TCR clonotypes was observed between tissues and blood (Fig. 3i), which is consistent with the tissue residency properties of T_RM_ cells. We then looked at T cell expansion in the EAT between patients with AF and patients in SR. Supporting the observed T_RM_ cell enrichment in patients with AF, clonal expansion was greater in the EAT of patients with AF compared to patients in SR, and diversity was reduced (Fig. 3j,k and Extended Data Fig. 3h,i). Together, these data support the notion that EAT acts as a reservoir of T_RM_ cells, which, upon activation, can migrate to the underlying myocardium to exert their function.

### CD8^+/−^ T_RM_ cells are transcriptionally diverse

**Fig. 4:**
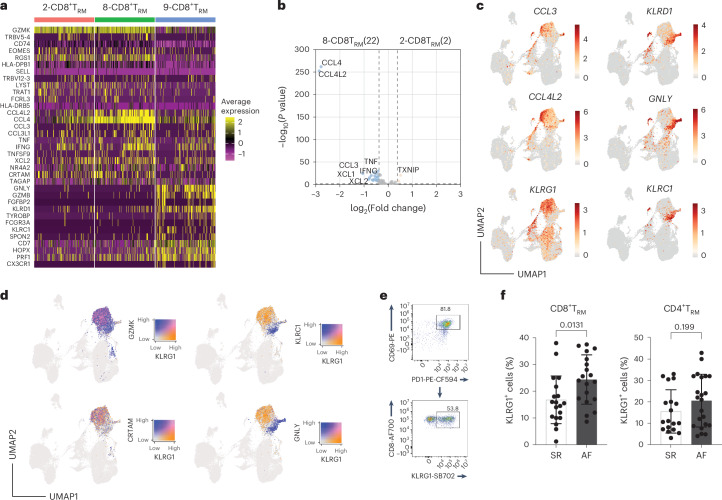
CITE-seq identifies two CD8^+^ T_RM_ cell populations with a distinct core set of genes. **a**, Heatmap shows average gene expression by curated CD8^+^ T_RM_ cell populations that had a fold change greater than 2 and *P* < 0.05 by the binomial test for at least one of the clusters. **b**, Volcano plots showing the average log fold changes and average Benjamini–Hochberg-corrected *P* values for pairwise differential expression between CD8^+^ T_RM_ cell clusters 8 and 2 based on the non-parametric Wilcoxon rank-sum test. **c**, UMAP of representative selected genes differentially expressed between two main CD8^+^ T_RM_ cell clusters. Color bars indicate average expression. Expression values are normalized for quantitative comparison within each dataset. **d**, UMAP showing co-expression of selective genes differentially expressed between two main CD8^+^ T_RM_ cell clusters. Color bars indicate level of overlap expression. **e**, Representative dot plot showing KLRG1 expression. **f**, Bar graphs indicate the frequency of KLRG1^+^CD4^+^ and CD8^+^ T_RM_ cells in the EAT. Each point represents an individual patient (*n* = 22 SR and *n* = 18 AF). Statistical significance was determined using two-tailed unpaired *t*-test for the parametrically distributed groups. Data are represented as mean and s.d.

Unsupervised clustering of CITE-seq data revealed a heterogeneous CD8^+^ T_RM_ cell population. To further characterize the CD8^+^ T_RM_ cells, DEGs between the three clusters were analyzed. Most of the DEGs were detected in cluster 9 compared to clusters 2 and 8, with clusters 2 and 8 differing only in the level of expression of effector molecules, such as *CCL4*, *CCL3* and *IFNG* (Fig. 4a,b and Supplementary Tables 7 and 8). Based on this, we concluded that clusters 2 and 8 are phenotypically similar populations in a different activation state. In contrary, cluster 9 appears to comprise a distinct CD8^+^ T_RM_ cell population with differential expression of NK receptors and cytotoxic molecules (Fig. 4a and Supplementary Table 7). Two similar populations were described in human intestinal tissue, which could be differentiated by the expression of KLRG1 (refs. ^28,29^). With clusters 2 and 8 exhibiting a more cytotoxic/activated phenotype, we then investigated if these CD8^+^ T_RM_ subsets were modulated in AF. We selected KLRG1 expression to assess CD8^+^ T_RM_ heterogeneity, as this marker is more highly expressed in clusters 2 and 8 (Fig. 2d); shows little overlap with genes expressed in cluster 9 (Fig. 4d); and can easily distinguish two populations by flow cytometry (Fig. 4e). We found that KLRG1^+^CD8^+^ T_RM_ cells were elevated in the EAT of patients with AF compared to patients in SR (Fig. 4f). Although a causal relationship cannot be established, these results suggest that an increase in KLRG1^+^CD8^+^ T_RM_ cells could signal local atrial inflammatory activation in patients with AF.

### Regional tissue remodeling in the EAT

**Fig. 5:**
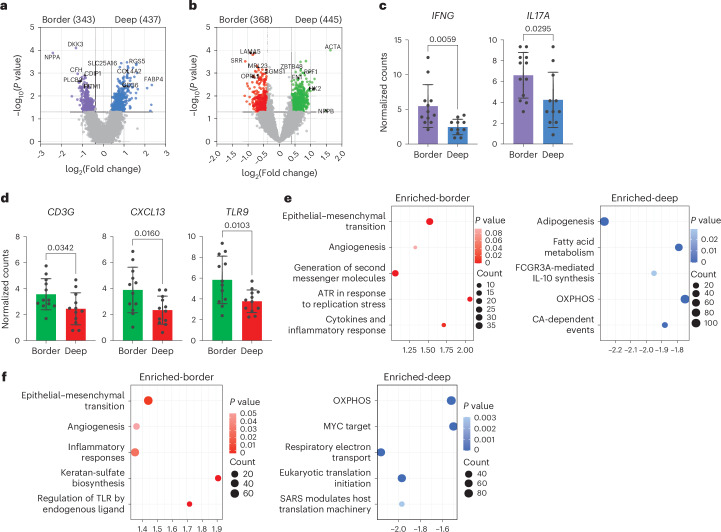
Regional differences identified by spatial transcriptomics. **a**, Volcano plot showing the average log fold changes in gene expression between border zone regions and deep in the tissue in the EAT. **b**, Volcano plot showing the average log fold changes in gene expression between border zone regions and deep in the tissue in the AA. **a**,**b**, Differential expression was performed using the linear mixed-effect model showing the average log fold changes and *P* values. **c**,**d**, Bar graph showing normalized counts of selective genes in the EAT (**c**) and AA (**d**), respectively. Statistical significance was determined using two-tailed paired *t*-test for parametric data, represented as mean and s.d. For normalized counts, Q3 normalization uses the top 25% of expressers to normalize across ROIs/segments **e**, GSEA pathway enrichment analysis of upregulated and downregulated DEGs in the EAT border zone compared to deep in the tissue. **f**, As in **e** but upregulated and dowregulated DEGs in the AA border zone compared to deep in the tissue. Pathway statistical significance was assessed using one-sided Fisher’s exact test. **a**–**e**, Assays were performed in three biological replicates in technical triplicates.

**Extended Data Fig. 4:**
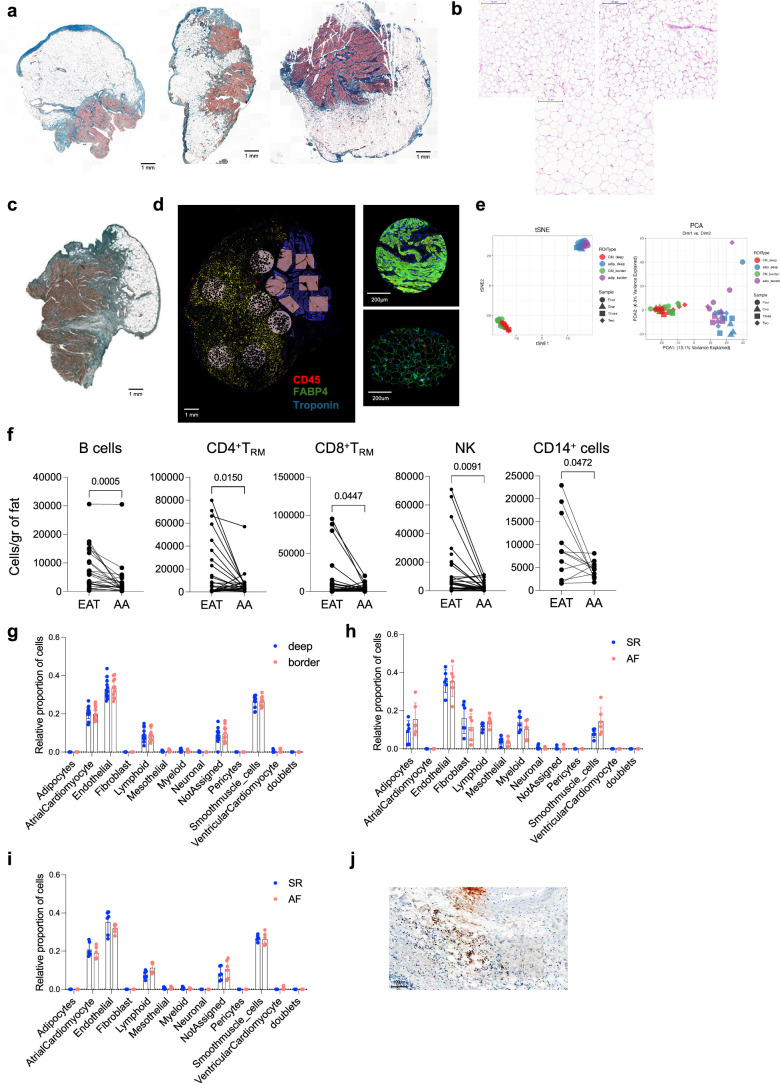
Spatial transcriptomics. **a**. Representative Masson’s Trichrome stains on tissue atrial specimens from 3 patients. **b**. Representative H&E stains on EAT specimens from 3 patients. **c**. Representative Masson’s Trichrome stain on tissue specimen at 2.0x magnification with the blue colour depicting the collagen deposition in the EAT/RAA interface and red-brown the atrial tissue from 3 patients. **d**. Representative immunofluorescent staining used for the identification of regions of interest (ROI) for spatial transcriptomic analysis. Cardiomyocytes were identified by troponin staining (blue), adipocytes by FABP4 expression (green) and immune cells by CD45 (red). **e**. Dimensionality reduction plots demonstrating sample clustering by type of tissue. **f**. Absolute number of immune cells in paired EAT and AA tissue samples. Statistical significance was determined by paired two-tailed T test, and data are represented as mean± SD (n=18 biological replicates). **g**. Bar graph comparing the proportion of cell types over total populations between the AA borderzone and deep in the tissue. **h-i**. Bar graph comparing the proportion of cell types over total populations between the EAT (**h**) or AA (**i**) borderzoneand deep in the tissue between patients with AF and on SR. **g-h**. Statistical significance was evaluated by two-way ANOVA with Sidak’s multiple comparison test. Bars represent mean±SD. **g-i**. Assays were performed in 3 biological replicates in technical triplicates. **j**. Representative immunohistochemistry staining of CCL5.

AF is characterized by structural remodeling of the atrial myocardium, which generally involves fibrotic changes in the atria^30^. EAT has been proposed as an important factor involved in structural and electrical remodeling, with recent work showing morphological changes in the EAT/atrial border zone^31,32^. Clusters of inflammatory cells were identified in the transition zone between adipocytes and fibrosis in the human atrium, with T cells being the dominant cell type^33^. The presence of tertiary lymphoid structures was not evident in our cohort (Extended Data Fig. 4a,b). To examine biological changes and regionality dictating structural remodeling in AF, we performed NanoString GeoMx Digital Spatial Profiling (DSP) of tissue biopsies from two patients with AF and two patients in SR. The list of genes tested is shown in Supplementary Table 9. For this regional transcriptional analysis, samples from deep in the tissue and at the EAT/AA border zone were used as shown in Extended Data Fig. 4c,d. To identify regional differences between the EAT and atrial tissue, we performed DEG analysis between regions from the same tissue. As expected, t-distributed stochastic neighbor embedding (t-SNE) and principal component analysis (PCA) showed a distinct transcriptomic profile between the tissues (Extended Data Fig. 4e). In total, 780 genes were found to be differentially expressed between deep in the EAT compared to the border zone, with 813 genes found to be differentially expressed within regions in the atrium (Supplementary Tables 10 and 11). Volcano plots showing all the DEGs are shown in Fig. 5a,b. Genes associated with inflammation, including *IFNG* and *IL17A*, were upregulated in the border zone of the EAT (Fig. 5c), whereas *CD3G* was found to be upregulated in the atrium border zone as well as *CXCL13* and several Toll-like receptors (Fig. 5d). Pathway analysis identified upregulation of the epithelial–mesenchymal transition, angiogenesis and inflammation pathways in both EAT and cardiomyocyte border zone (Fig. 5e,f). A decrease in oxidative phosphorylation (OXPHOS) was detected in the EAT and AA border zone. A decline in mitochondrial OXPHOS activity was previously reported in chronic heart failure^34^.

**Fig. 6:**
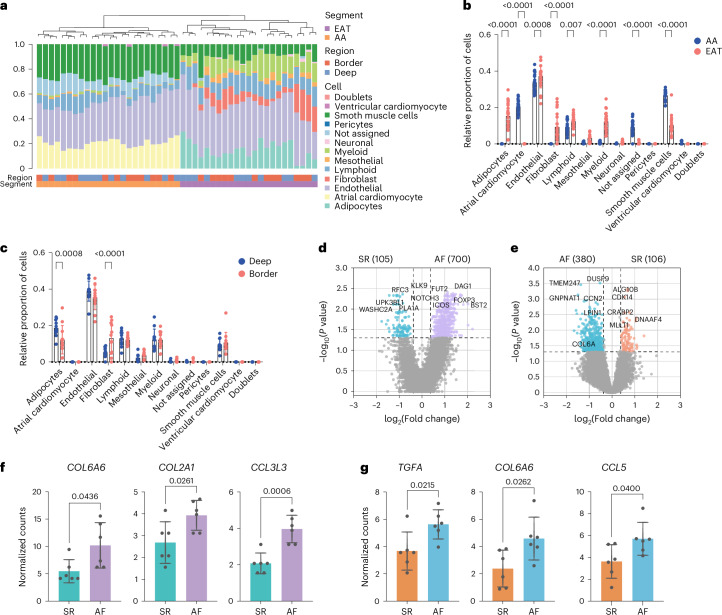
Tissue remodeling in the border zone. **a**, Proportion of cell types in the EAT and AA identified by cellular deconvolution. Each bar represents an individual ROI. **b**, Bar graph comparing the proportion of cell types over total cells between the EAT and AA. **c**, Bar graph comparing the proportion of cell types over total cells between the EAT border zone and deep in the tissue. **b**,**c**, Statistical significance was evaluated by two-way ANOVA with Sidak’s multiple comparison test. Bars represent mean ± s.d. **d**, Volcano plots showing the average log fold changes in gene expression in the EAT border zone between patients with AF and patients in SR. **e**, As in **d** but showing expression differences in the AA border zone between patients with AF and patients in SR. **d**,**e**, Differential expression was performed using the linear mixed-effect model showing the average log fold changes and *P* values. **f**, Bar graph showing normalized counts of selective genes in the EAT border zone between patients with AF and patients in SR. **g**, As in **f** but in the AA border zone between patients with AF and patients in SR. **f**,**g**, Statistical significance was determined using two-tailed paired *t*-test for parametric data, represented as mean and s.d. For normalized counts, Q3 normalization uses the top 25% of expressers to normalize across ROIs/segments. **a**–**g**, Assays were performed in three biological replicates in technical triplicates.

To investigate tissue remodeling, we performed a cellular deconvolution analysis. Cellular composition differs among tissues, with myeloid, lymphoid and mesothelial cells and fibroblasts being enriched in the EAT. As expected, adipocytes and atrial cardiomyocytes were exclusively present in the EAT and AA, respectively (Fig. 6a,b). Similarly, mesothelial cells were elevated in the EAT, probably due to the mesothelial lining of the heart. The EAT hosts the cardiac autonomic nerve fibers, the ganglionated plexi and a considerable amount of endothelial progenitor cells, explaining the elevated proportion of endothelial and neuronal cells (Fig. 6a,b). Consistent with the recognition of the adipose tissue as an immunological organ^35^, a proportion of monocytes and lymphocytes was elevated in the EAT. This was confirmed by flow cytometry (Extended Data Fig. 4f). When investigating regional intra-tissue differences, fibroblasts were more abundant in the EAT border zone, whereas adipocytes were increased deep in the tissue, which supports previous reports on decreased adipogenesis in the border zone^31^ (Fig. 6c). No differences were observed within the AA (Extended Data Fig. 4g).

We then investigated differences between the border zone of patients with AF and SR controls. Cellular deconvolution analysis showed a trend toward an increase in smooth muscle cells in the EAT of patients with AF, albeit not significant (*P* = 0.06) (Extended Data Fig. 3f,g). However, DEG analysis identified 700 and 380 genes being upregulated in the EAT and AA of patients with AF, respectively (Supplementary Tables 12 and 13). In addition, both EAT and AA showed upregulated fibrosis-related genes in patients with AF compared to SR controls (Fig. 6d,e). Similarly, inflammatory markers were upregulated in patients with AF (Fig. 6f,g). CCL5, which is highly expressed by T_RM_ cells, was upregulated in the AA border zone in AF (Fig. 6g and Extended Data Fig. 3h). Together, these data indicate that the inflammatory response in the intersection within tissues is accompanied by secretion of pro-fibrotic factors and cellular remodeling at least in the EAT, which is more evident in patients with AF.

### T_RM_ cells can directly modulate cardiomyocyte function

**Fig. 7:**
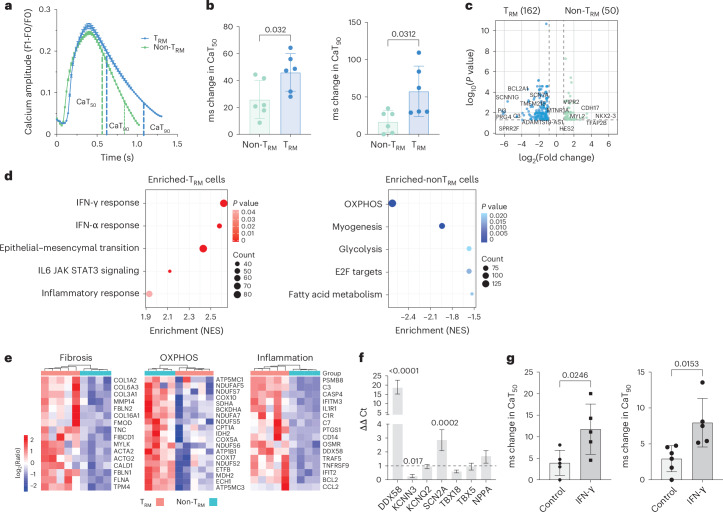
Calcium dynamics in iPSC atrial cardiomyocytes. **a**, Graph demonstrating a typical calcium transient after addition of T cells with the key parameters CaT_50_ and CaT_90_ depicted (*n* = 6 biological replicates). Each point represents mean ± s.e.m. **b**, Bar graphs demonstrating percentage change in CaT_50_ and CaT_90_ in CD4^+^ T_RM_ and non-T_RM_ cells (*n* = 6 biological replicates). Statistical significance was assessed using two-tailed *t*-test for parametric data, represented as mean and s.d. **c**, Volcano plot demonstrating differential gene expression between the T_RM_ and non-T_RM_ samples. Significance threshold of *P* < 0.05 log_10_ adjusted and log_2_ fold change > 1. **d**, GSEA pathway enrichment analysis of upregulated and downregulated DEGs in the iPSC-CMs cultured with CD4^+^ T_RM_ cells compared to non-T_RM_ cell cultures. Pathway statistical significance was assessed using one-sided Fisher’s exact test. **e**, Heatmap showing expression of genes associated with fibrosis, OXPHOS and inflammation. **c**,**e**, The Wald test from the DESeq2 package was used to test significance using false discovery rate-adjusted *P* values. **f**, iPSC-CMs were cultured with recombinant 50 ng ml^−1^ IFN-γ for 8 h. Relative expression levels of selective genes in iPSC-CMs (*n* = 6 technical replicates from three independent experiments) were analysed by RT–PCR. Expression levels were normalized to GAPDH expression. Bars represent expression in treated cells compared to untreated, which was set at 1 and indicated with dotted lines. Bars represent the mean ratio and upper and lower limits. Statistical significance was determined using unpaired two-tailed *t*-test. **g**, Bar graphs demonstrating percentage change in CaT_50_ and CaT_90_ in iPSC-CMs treated with IFN-γ (*n* = 5 technical replicates). Statistical significance was determined using unpaired two-tailed *t*-test for parametric data, represented as mean and s.d. NES, normalized enrichment score. Source data

**Extended Data Fig. 5:**
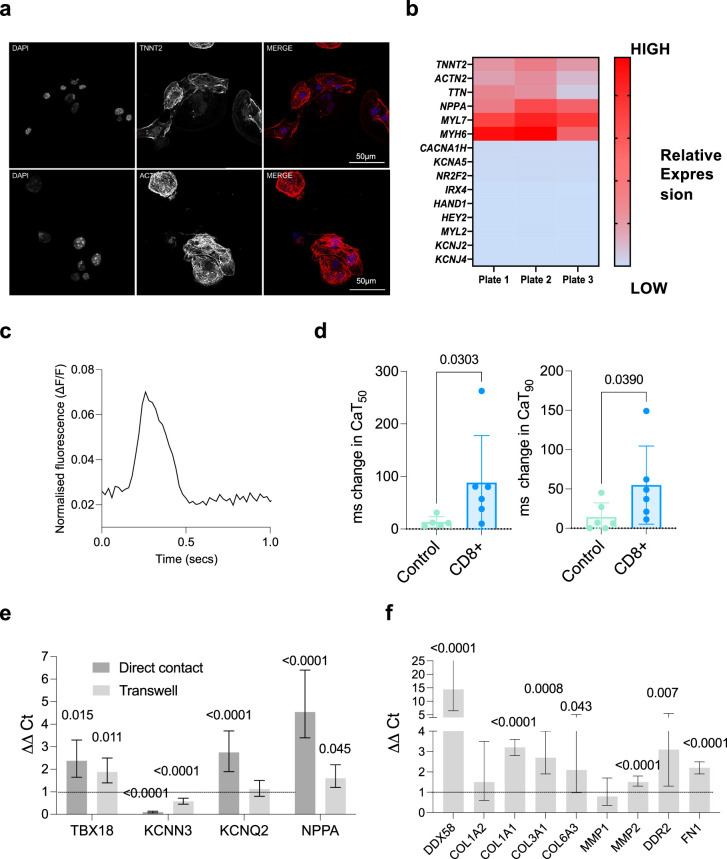
Characterisation of iPSC-derived atrial cardiomyocytes. **a**. Representative immunofluorescence images depicting DAPI (nuclear stain), troponin (TNNT2) and smooth muscle actinin (ACTN2), and the merged images (DAPI) in blue, TNNT2 and ACTN2 in red). Scale bar 50 μM (3 independent experiments). **b**. Heat map demonstrating the relative expression of key atrial cardiomyocyte genes in independently differentiated cardiomyocyte plates (n=3). **c**. Atrial cardiomyocyte action potential waveform recorded using the voltage-sensitive dye, di-4-ANEPPS. **d**. Bar graphs demonstrating percentage change in CaT_50_ and CaT_90_ in CD8^+^ T cells compared to untreated cells. Biological replicates n=6. Statistical significance was assessed using two-tailed t-test for parametric data, represented as mean and SD **e**. iPSC-cardiomyocytes were cultured directly with T_RM_ cells or in the presence of a 0.4 μM transwell were analysed by RT-PCR. Relative expression levels of selective genes in iPSC-cardiomyocytes (n = 6 biological replicates in triplicates) were analysed by RT-PCR. **f**. As in **e**, but bars represent expression of IFNγ−treated iPSC-cardiac fibroblast compared to untreated cells from three independently experiments (n = 9). **f-e**. Bars represent the mean ratio and upper and lower limits. Statistical significance was determined using unpaired two-tailed *t*-test. Source data

To evaluate the hypothesis that T_RM_ cells can significantly alter the electrical properties of coupled cardiomyocytes compared to non-T_RM_ cells, we performed co-culture studies with induced pluripotent stem cell–derived cardiomyocytes (iPSC-CMs) with an atrial phenotype. Atrial cardiomyocytes were differentiated as described by Cyganek et al.^36^. Contracting cardiomyocytes could be visualized around day 8 after differentiation, with spontaneous and consistent beating cell sheets evident after further maturation (Extended Data Fig. 5a–c). The cardiomyocyte phenotype was confirmed by transcriptomic, proteomic and electrophysiological analysis (Extended Data Fig. 5). Calcium is a fundamental link between the electrical activity in the heart and contractility of the cardiomyocytes. Changes in the calcium transient occur dynamically throughout the course of cardiomyocyte contraction, whereas perturbations in calcium flux are associated with arrhythmia vulnerability^37^. Co-culture of iPSC-CMs with CD4^+^ T_RM_ cells isolated from EAT significantly altered calcium transient parameters at 50% (CaT_50_) and 90% (CaT_90_) decay in cellular calcium handling compared to co-cultures with non-T_RM_ memory CD4^+^ T cells (Fig. 7a,b). The low frequency of non-T_RM_ memory CD8^+^ T cells precluded collection of sufficient cells to assay their effects on cardiomyocyte calcium handling. However, similar changes in calcium flux were observed with total CD8^+^ T cells (Extended Data Fig. 5d).

To determine whether transcriptomic alterations accompanied changes observed in calcium handling, we performed RNA-seq analysis of iPSC-CMs isolated after co-cultures. DEG analysis revealed an upregulation of gene-encoding ion channels—for example, *SCNN1*, *SCN7A*, *SCN2A*, *KCNQ2* and *KCNN3*—in iPSC-CMs co-cultured with CD4^+^T_RM_ cells compared to non-T_RM_ cells, with *KCNN3* and *SCN2A* previously associated with AF^38,39^ (Fig. 7c and Supplementary Table 14). Interestingly, several extracellular matrix and collagen genes were upregulated in T_RM_ cell co-cultures, including *HAS1*, *PAPLN*, *FMOD*, *COL3A1*, *COL6A3* and *COL1A2*. Similar collagen expression was reported in iPSC-CMs under fibrotic stiffness^40^. Genes associated with apoptosis, such as *CASP4* and *BCL2*, and complement activation, for example, *C3*, *C7* and *C1R*, were also upregulatef on iPSC-CM-T_RM_ co-cultures. Gene set enrichment analysis (GSEA) identified an enrichment of genes associated with epithelial–mesenchymal transition and angiogenesis in T_RM_ cell co-cultures, and OXPHOS, adipogenesis and fatty acid metabolism were upregulated in iPSC-CMs co-cultured with non-T_RM_ memory CD4^+^ T cells, which is consistent with regional differences detected with spatial transcriptomic analysis (Fig. 7d,e and Supplementary Table 14). Inflammatory pathways, such IFNs and inflammatory responses, were upregulated in iPSC-CM-T_RM_ co-cultures. This is likely a response to the enhanced production of pro-inflammatory cytokines and cytotoxic factors by T_RM_ cells (Fig. 7d). To test if cytokines alone could modulate iPSC-CMs, we analyzed selective gene expression changes in co-cultures performed in the presence of a 0.4-μm transwell. Expression of genes, such as *NPPA* and *KCNN3*, was modulated by T_RM_ in both conditions, albeit at a higher level with direct cell-to-cell contact, whereas *KCNQ2* expression was upregulated only by direct contact (Extended Data Fig. 5e). *IFNG* expression is upregulated in the atria of patients with AF, and plasma IFN-γ has been described as an independent risk factor for all-cause mortality in AF^41,42^. IFN-γ is highly produced by T_RM_ cells, and its signaling pathway was upregulated in cardiomyocyte co-cultures. Thus, we then tested if IFN-γ alone could modulate iPSC-CM function. IFN-γ upregulates *DDX58* expression, consistent with the upregulation of the IFN-γ pathway, and was sufficient to modulate ion channel expression, such as *KCNN3* and *SCN2A*, and cellular calcium handling compared to untreated cells (Fig. 7f,g). IFN-γ production by T cells has been shown to modulate cardiomyocyte and cardiac fibroblast gene expression^43^. Indeed, IFN-γ stimulation of cardiac fibroblasts upregulated the expression of DDR2 and collagen genes (Extended Data Fig. 5f). Overall, our data suggest that T_RM_ cells can promote AF by several mechanisms.

## Discussion

EAT has emerged as a risk factor and independent predictor of AF incidence and recurrence after ablation^44^. Inflammation has been described as a possible mechanism driving this increased risk. However, in-depth investigations of EAT immune profiling remain sparse. We and others have shown that EAT is highly enriched in adaptive immune cells, in particular T cells^19,45^. In the present study, we found that T_RM_ cells, which comprise a distinct subset of memory T cells in tissue, were significantly elevated in the EAT of patients with AF. These cells showed a high degree of expansion and migration toward the atrial tissue and can directly impair cardiomyocyte calcium handling. Furthermore, a highly heterogenous spatial organization is present within atrial border zones, implying a mechanism by which EAT may cause the non-uniform conduction disturbances observed in human AF.

T_RM_ cells have superior effector functions, including rapid chemokine/cytokine production and cytotoxicity^20^, providing local protective immune responses against pathogens. More recently, however, it was demonstrated that T_RM_ cells can mediate autoimmunity, for example in inflammatory skin conditions, arthritis and Crohn’s disease^29,46,47^. The role of T_RM_ cells in cardiovascular diseases, in particular AF, has not been explored. Here we demonstrated that the presence of T_RM_ cells positively correlates with production of IL-17 and IFN-γ, both known to be implicated in AF risk^48^. Furthermore, CITE-seq analysis identified heterogeneity within the CD8^+^ T_RM_ cell pool. KLRG1 expression was used to define two main CD8^+^ T_RM_ populations with distinct effector functions. Interestingly, these two subsets closely resemble the ones described in the context of intestinal inflammation^28,49^, with KLRG1^+^ T_RM_ cells identified by gene expression of *GZMK*, *GZMH*, *CCL4*, *CRTAM* and *KLRG1*, and a second population expressing CD103 was positive for *CD7*, *KLRB1* and *CAPG* expression, among others^49^. Of note, CD103 expression is limited to CD8^+^ T_RM_ cells at mucosal sites and, therefore, was not expressed in T_RM_ cells in the EAT and AA^24^. Although CD103^+^ T_RM_ cell frequency has been noted in healthy intestines, KLRG1^+^ T_RM_ cells showing enhanced cytotoxic and proliferative potential were elevated in Crohn’s disease^29^. These findings were later confirmed in a mouse model of intestinal infection^50^. In this model, CD103 fate mapping identified CD103^−^ intestinal T_RM_ cells as the first responders to secondary infection. These cells were more frequently in contact with CD11c^+^ dendritic cells in tissue and exhibited in situ proliferation, enhanced reactivation and effector function potential^50^. The association of KLRG1 expression with T_RM_ cell pro-inflammatory phenotype is particularly relevant given that the proportion of KLRG1^+^CD8^+^ T_RM_ cells was higher in patients with AF. Additional work is required to establish a direct link between the presence of KLRG1^+^CD8^+^ T_RM_ cells in the tissue and AF pathogenesis, but KLRG1^+^ T_RM_ cells have the potential to serve as a predictive biomarker of disease persistence and/or recurrence.

It is worth noting the constitutive high expression of PD1 on T_RM_ cells^51,52^. PD1 is an inhibitory receptor, with its expression traditionally associated with T cell exhaustion. However, recent findings point toward an involvement of PD1 in restraining T_RM_ cell activation and immunopathology. Indeed, chronic pancreatitis is associated with reduced PD1 expression, and inhibition of the PD1/PDL1 axis resulted in enhanced T_RM_ cell-mediated functional responses^51^. Consistently, the presence of PD1^+^ T_RM_ cells in tumors is associated with good prognosis and increased T cell effector capacity after anti-PD1/PDL1 immune checkpoint inhibitor (ICI) treatment^53,54^. However, PD1 blockade can also lead to cardiac arrhythmias in patients with cancer^49,55^. Moreover, the frequency of active episodes in patients with AF correlates with lower surface expression of PD1/PDL1 on peripheral CD4^+^ T and dendritic cells^56^. Further investigation is required to understand the risk factors and to establish a link between T_RM_ cell activation and ICI-associated arrhythmias in these individuals.

EAT acts as a rich local depot of vasoactive molecules, cytokines and growth factors that can act on the heart or be secreted via the vaso vasorum into the coronary vessels (which supply both the EAT and myocardium) to exert its effects^12^. The EAT secretome varies considerably in physiological and pathophysiological states, with numerous lines of evidence implicating EAT inflammation as a key player in AF pathogenesis^12,16^. The EAT secretome applied to ex vivo atrial explants was found to induce fibrosis, a hallmark of AF, with transformation of fibroblasts into myofibroblasts and the production of a large amount of extracellular matrix components^57^. In vitro cultures with cardiomyocytes resulted in electrophysiological changes and electrical remodeling of cardiomyocytes^58^. Less is known about the direct role of immune cells and immune cell migration between tissues. Adipose tissue is increasingly recognized as an accessory immune organ contributing to immune responses. Fat-associated lymphoid clusters (FALCs) have been identified in several adipose tissue depots in mice and humans, which are not encapsulated organs, akin to tertiary lymph nodes supporting B cell responses^59^. In mice, adipose tissue has been shown to represent a memory T cell reservoir that provides rapid effector memory (EM) responses. These T cells were predominantly T_RM_ cells that expanded in situ and were redeployed to adjacent tissues to confer protection against secondary infections^25^. Our flow cytometry and spatial transcriptomic data support this idea that EAT is an immune reservoir where myeloid and lymphoid cells are present at higher numbers than in the heart tissue. This numeric advantage can be the result of a bioenergetic rich environment provided by the EAT and/or protective mechanism to restrain the accumulation of adaptive immune cells in the heart.

EAT/AA has emerged as a hotspot of fibrosis and cellular infiltration with large amounts of collagen deposition^31,32^. Due to the anatomical contiguity between EAT and AA, EAT may be a key driver of the fibrotic milieu, as evidenced by the accumulation of fibroblasts and upregulation of inflammatory and extracellular matrix components in the EAT border zone, which was exacerbated in patients with AF. The absence of cellular differences within the AA was surprising, in particular the low proportion of fibroblasts compared to EAT. Notably, many key highly expressed genes are shared between the collagen-producing myofibroblast population and smooth muscle cells, such as *MYH11* and *ACTA2*, suggesting that these cells may have not been adequately distinguished by the deconvolution algorithm. In addition, the deconvolution analysis did not allow for the identification of lymphoid cell subsets. The regional differences could be explained, at least in part, by inter-tissue and intra-tissue T_RM_ cell migration, supported by the dynamic relationship observed among T_RM_ TCR clones. T cell expansion was predominately detected among T_RM_ cells showing a high degree of shared clonality, highlighting the local immune crosstalk between the overlying adipose tissue (EAT) and the myocardium (AA). The finding that T_RM_ cells in general, and TCR-expanded clones in particular, have a more activated phenotype in the AA compared to EAT suggests a migration process from the reservoir in the EAT toward the effector site in the AA. Similar findings have recently been described in the context of heart failure^60^. High shared clonality could be detected between the EAT and the heart, with T cells having a more activated phenotype in these patients. This is no surprise given that AF and heart failure have common pathophysiological mechanisms^61^, with EAT dysfunction and inflammation thought to play an instrumental role in both disease processes.

Relative to patients in SR, the number of T cells has been shown to be elevated in the atrial tissue of patients with AF^62^, with an early study highlighting an accumulation of CD8^+^ T cells at the EAT/AA tissue border zone^33^. However, how T cells can directly impair atrial conduction and function is not established. T cells are key mediators of tissue inflammation, which can alter atrial electrophysiology. Inflammatory cytokines, such as TNFα, IL-1β and IL-17, can markedly enhance the risk of arrhythmic events by directly promoting electrical and structural cardiac remodeling^48^. In addition, IFN-γ has been shown to exert a sustained inhibitory effect on cardiac L-type calcium channels^63^ and to induce a metabolic shift in cardiomyocytes with downregulation of OXPHOS, which is consistent with the failing heart^43^. We showed that CD4^+^ T_RM_ cells were able to uniquely alter the calcium handling properties of atrial cardiomyocytes by inducing electrical and structural changes as evidenced by gene expression changes in ion channels, calcium signaling, tracellular matrix and collagen. This is consistent with the high production of pro-inflammatory cytokines by T_RM_ cells—for example, TNF-α and IFN-γ—although a direct cell-to-cell contact, for example by NK receptor binding, cannot be ruled out. T_RM_ cells also express a cluster of chemokines and chemokine receptors, with CCL5 emerging as a possible mediator of tissue inflammation. CCL5 mediates trafficking and homing of T cells and innate cells to sites of inflammation. CCL5 is mainly expressed by T cells and monocytes, although CITE-seq analysis identified T_RM_ cells as the main producers of CCL5 in the EAT and AA, with CCL5^+^ cells in the border zone showing a clear lymphocyte morphology. Blocking of CCL5 significantly reduced infarct size in mouse models of heart failure and was identified as a key inflammatory mediator in the EAT of patients with heart failure^60,64^. Furthermore, production of CCL5 by T_RM_ cells is thought to be responsible for arthritis flares by promoting the recruitment of T_EM_ cells to the joint^47^.

An important limitation of this study is that, due to the nature of the tissue analyzed, samples were obtained from patients with heart conditions that may themselves alter the physiology of the EAT and AA. Although the presented data provide evidence of a strong association between T_RM_ cell-induced inflammation and increased susceptibility to AF, a causative relationship was only partially confirmed by in vitro iPSC cultures. A limitation of the iPSC-CM system is that T cell exposure to self-antigens that may be present in patients with AF is limited. In addition, it is likely that the effect of T_RM_ cells in cardiomyocytes was underestimated, as the outcome of a positive feedback loop effect—for example, recruitment of innate and adaptive immune cells to the site of inflammation—can be assessed only in the presence of a full immune system.

## Methods

### Study population and sample collection

All patients provided written informed consent for their participation in the study as per local research procedures and Good Clinical Practice guidance (Research Ethics Committee reference: 14/EE/0007). Adult (≥18 years) patients undergoing on-pump open chest coronary artery bypass grafting (CABG) surgery and/or valve reconstruction (VR) surgery were recruited from Barts Heart Centre, St. Bartholomew’s Hospital, in London, United Kingdom (UK), via the Barts BioResource. Exclusion criteria included congenital heart disease; underlying cardiomyopathies or ion channelopathies; primarily undergoing other cardiac surgical procedures (for example, aortic surgery); off-pump CABG surgery; patients with active endocarditis, myocarditis or pericarditis; patients with pre-existing inflammatory diseases (for example, rheumatoid arthritis); active malignancy; patients on immunomodulatory or biologic drugs (for example, tacrolimus and anti-TNF-α agents); perioperative rhythm control therapies (for example, use of amiodarone); postoperative hemodynamic shock; uncorrected potassium derangement (K < 3.3 or K > 5.8); and uncorrected magnesium derangement (Mg < 0.5 or Mg > 1.5) detected on laboratory blood sample analysis. Fasting venous blood samples were collected preoperatively in the anesthetic room. Approximately 0.8–1 g of adipose tissue samples was collected in ice-cold PBS with 2% FBS. SAT was collected immediately after the median sternotomy incision, and EAT was obtained after opening up of the pericardial sac. AA tissue was obtained after insertion of the right atrial cannula as part of transitioning patients on to cardio-pulmonary bypass, and, typically, 0.1–0.5 g of AA tissue was harvested.

### Sample processing

Adipose tissue samples were processed as previously described^65^. AA samples were enzymatically digested with 675 U collagenase I (Sigma-Aldrich), 187.5 U collagenase XI (Sigma-Aldrich) and 10 U DNase (Sigma-Aldrich) in 1 ml of HBSS modified with 10 mM HEPES but without phenol red (STEMCELL Technologies) per gram of tissue. The cell–enzyme suspension was incubated at 37 °C with 225-r.p.m. agitation for 45 min. Then, 5 ml of fasting venous blood samples was collected in EDTA tubes (BD Biosciences) preoperatively in the anesthetic room. Peripheral blood mononuclear cells (PBMCs) were isolated using Ficoll-Paque PLUS (Cytiva) as per the manufacturer’s instructions. Single-cell suspensions were obtained after centrifugation and red cell lysing before antibody staining.

### Flow cytometry

Immune cells from blood, adipose tissue and AA tissue were isolated as described previously. Immune cells were stained with fixable Aqua Live/Dead cell stain (Invitrogen) diluted 1:1,000 and fluorochrome-conjugated antibodies specific for CD197-FITC, CD19-PerCP-Cy5.5, CD45RO-BV421, CD335-BV605, CD45-BV785, CD127-APC, CD8-AF700, CD3-APC/Cy7, CD69-PE, CD4-PE/Cy7, PD1-PE-CF594, CD303-FITC, CD123-PerCP/Cy5.5, CD206-BV421, CD3-BV605, CD19-BV605, CD14-APC, CD16-AF700, CD1c-APC/Cy7, Clec9A-PE, CD1a-PE-CF594 and CD141-PE/Cy7 from BioLegend and KLRG1-SB702 from eBioscience. The samples were stained at 4 °C for 18 min and then washed twice with fluorescence-activated cell sorting (FACS) buffer where the plate was centrifuged at 400*g* for 3 min and the supernatant discarded. The samples were then fixed in stabilizing fixative buffer (BD Biosciences) containing 3% paraformaldehyde at 4 °C for 30 min.

For intracellular cytokine staining, samples were resuspended in 500 μl of Aim V medium with the addition of 1 μl of Cell Activation Cocktail (BioLegend). After 4-h incubation, samples were washed and stained for surface markers as detailed above, followed by permeabilization and fixation in the permeabilization/fixation buffer (BD Biosciences) at 4 °C for 12 min. Intracellular cytokine production was evaluated by incubation for 15 min at 4 °C with fluorochrome-conjugated antibodies specific for IFN-γ-APC, IL-17-APC/Cy7 and IL-22-PE (BioLegend). Data were acquired on a CytoFLEX (Beckman Coulter) and analyzed using FlowJo version 10 software.

### CITE-seq and single-cell TCR sequencing

Paired AA and EAT samples were collected from two patients with AF (1× VR and 1× CABG) and digested as outlined above. The CITE-seq samples were prepared following the steps outlined in the 10x Genomics Cell Surface Protein Labeling for Single Cell RNA Sequencing protocols with the Feature Barcode technology protocol preparation guide (document CG000149). In brief, samples were resuspended in 50 μl of PBS + 1% BSA and 5 μl of human TruStain FcX and incubated for 10 min at 4 °C. Fixable Aqua Live/Dead cell stain (Invitrogen) and CD45 PE antibodies (BioLegend) and TotalSeq antibodies were resuspended in PBS and added to the sample suspension to a create a total sample volume of 155 μl. The following TotalSeq antibodies (BioLegend) were employed: C0138 anti-human CD5, C0358 anti-human CD163, C0160 anti-human CD1c, C0049 anti-human CD3, C0072 anti-human CD4, C0080 anti-human CD8a, C0087 anti-human CD45RO, C0148 anti-human CD197, C0146 anti-human CD69, C0088 anti-human CD279 and C1046 anti-human CD88. Samples were then incubated at 4 °C for 30 min in the dark. After washing, samples were incubated for 15 min with MojoSort human anti-PE Nanobeads followed by magnetic purification as per the manufacturer’s instructions (BioLegend) for CD45^+^ enrichement. Live CD45^+^ cells were further purified by FACS with an LSRFortessa analyzer (BD Biosciences).

### The 10x Genomics CITE-seq

Chromium Next GEM Single Cell 5′ v2 (dual index) with Feature Barcode technology for Cell Surface Protein & Antigen Specificity User Guide (CG000330) was employed at the UCL Single-Cell Sequencing facility. In brief, the cell suspension was partitioned into a nanoliter-scale droplet emulsion using the 10x Genomics Chromium Single Cell Controller with RNA-seq libraires created using the Chromium Next GEM Single Cell 5′ Reagent Kits and a Gel Bead Kit v2. Gel beads in emulsion (GEM) were generated by combining the barcoded Single Cell VDL 5′ gel beads with a master mix containing the cell surface protein-labeled cells and partitioning oil onto a Chromium Next GEM chip. The gel beads were dissolved, and the cells were lysed. After reverse transcription, barcoded full-length cDNA was generated from the poly-adenylated mRNA. Silane magnetic beads (Dynabeads MyOne SILANE) were used to purify the 10x barcoded cDNA. Libraries were then sequenced in-house at the UCL Single-Cell Sequencing facility on an Illumina NextSeq 500/550 sequencing platform.

### CITE-seq data processing

CITE-seq data processing was performed at the UCL City of London Centre Single-Cell Sequencing core. In brief, output from the Chromium Single Cell 5′ v2 sequencing was processed using Cell Ranger (version 6.0.1) analysis pipelines. FASTQ files were generated using Cell Ranger mkfastq (version 6.0.1). Gene expression reads were aligned to the human reference genome GRCh38 and counted using Cell Ranger count (version 6.01.) VDJ reads were aligned to the GRCh38 VDJ reference dataset using Cell Ranger vdj (version 6.01). 10x feature barcoding was performed using the antibodies outlined above, the reads for which were counted using Cell Ranger count.

Expression matrices were analyzed using the Seurat package (version 4.0.03) in R. Cells with mitochondrial reads making up more than 10% of the total read count, or with fewer than 400 genes detected, were removed. A multiplet filtering step using DoubletFinder (version 2.0.3) was performed using the author-recommended settings. Normalization was performed on the dataset using SCTransform (version 0.3.2) using centered log-ratio normalization and the top 3,000 variable genes. This was followed by Seurat (version 4.0.3) integration to remove batch effects, and the top 3,000 genes minus TCR genes were used as integration features. PCA and UMAP dimensionality reduction (dims 1:30) was performed using RunPCA and RunUMAP from the single-cell RNA-seq data only. Clustering was performed using the FindClusters function in the Seurat package using a resolution of 0.8. All differential gene expression analysis was carried out on log-normalized gene expression values using the Seurat NormalizeData function with default parameters through the MAST algorithm within FindMarkers. Feature barcoding reads were normalized using a centered log-ratio transformation. VDJ data were integrated using strict clone calling—matching VDJC gene and TRA/TRB nucleotide sequences. Analysis was performed using scRepertoire (version 1.3.5) and STARTRAC (version 0.1.0).

### Bulk TCR sequencing

Bulk TCR α and β sequencing was performed from paired blood AA and EAT samples from five patients with AF. In addition, TCR sequencing was performed using additional EAT from five patients in SR. cDNA was extracted from tissues and whole blood. A quantitative experimental and computational TCR sequencing pipeline was employed as previously described^66^. The pipeline introduces unique molecular identifiers (UMIs) attached to individual cDNA TCR molecules, allowing correction for PCR and sequencing errors. TCR identification, error correction and CDR3 extraction were performed following a suite of tools available at https://github.com/innate2adaptive/Decombinator, as detailed in Peacock et al.^66^. TCR frequency and similarity were analyzed using the Immunarch package in R (version 1.0.0). The level of similarity between the different TCR repertoires was measured using the Morisita–Horn index, ranging from 0 (no similarity) to 1 (identical), which takes into account the shared sequences and clonal frequency between samples. Antigen matching analysis was performed via the McPAC-TCR database.

### Spatial transcriptomics

Spatial profiling was carried out by NanoString Technologies using a GeoMx DSP. AA tissue with surrounding EAT samples was selected from four patients, of which two were from patients with AF and two were from patients in SR. The technology is based on the principle of in situ hybridization. After dewaxing of the formalin-fixed, paraffin-embedded (FFPE) slides, Tris-EDTA buffer was added to expose the RNA targets, and a proteinase K digestion was performed to remove any protein bound to RNA. The tissue was incubated overnight at 37 °C with GeoMx RNA detection probes. Labeled antibodies, FABP4 for adipocytes and troponin for cardiac tissue were added to image the tissue. The GeoMx DSP uses an automated microscope to image the tissue sections and cleave/collect the photocleavable indexing oligonucleotides. Specific regions of interest (ROIs) were then pre-selected on either side of the border zones of the AA/EAT and deep in the AA and deep in the EAT in triplicates. ROIs were quantified using RNA-seq technology on the Illumina platform to generate RNA expression data within a spatial context.

### Spatial transcriptomics data processing

Data were analyzed with the GeoMx DSP Analysis Suite. The first quality control step looks at the ‘raw read threshold’ flagging segments with fewer than 1,000 raw reads. A second step assesses the percentage of aligned reads. The sequenced barcodes should match the known GeoMx library of barcodes; hence, a high percentage alignment should be expected (an alignment threshold value less than 80% is typically used to flag segments). Sequencing saturation was assessed as 1 − (aligned reads/de-duplicated reads). A value of less than 50% sequencing saturation was used to flag segments. A background quality control step was additionally performed based on negative probes. A no-template control was included in the first well of each collection plate with the negative control in PCR reading very low counts. The Grubbs outlier test is performed to exclude a probe from all segments if the probe is higher or lower in more than 20% of segments. A limit of quantitation value is also defined where a target is considered to be detected with high confidence, the default setting here being 2 standard deviations above the geometric mean of the negative probes. Filtering was performed to further refine the dataset, followed by Q3 normalization.

For spatial deconvolution, the SpatialDecon plugin in R version 1.2 (NanoString Technologies) was employed. The human heart benchmarking cell matrix used for the alignment of the GeoMx spatial gene expression data was extracted from the single cell data of the human heart (heartcellatlas.org). The deconvolution script with the adjusted cell matrix was run on the GeoMx DSP Analysis Suite applied to the normalized spatial gene expression data.

### iPSC atrial cardiomyocyte and cardiac fibroblast differentiation

iPSC lines were acquired from the Human Induced Pluripotent Stem Cell Initiative (HipSci) deposited by the Wellcome Trust Sanger Institute into the Culture Collections archive (UK Health Security Agency). Each cell line was resuscitated as per the HipSci guidance (https://www.culturecollections.org.uk/media/109442/general-guidelines-for-handling-hipsci-ipscs.pdf) in TeSR-E8 media (STEMCELL Technologies). Plates were coated with a vitronectin coating (Thermo Fisher Scientific) to provide an appropiate adhesive surface for culturing iPSCs. Rho kinase (ROCK) inhibitor RevitaCell (Thermo Fisher Scientific) was added to TeSR-E8 medium to achieve a final concentration of 10 μM (1:1,000 dilution from the stock solution). Cells were plated in 2 ml of TeSR-E8 RevitaCell media per well in a six-well plate. iPSC media were replaced daily with fresh TeSR-E8. Cells were typically split at a ratio of 1:4 every 6–7 d, following HipSci guidance. iPSC-CM derivation was carried out as per Cygnaek et al.^36^. The base cardiomyocyte differentiation medium (CDM) used was RPMI 1640 with HEPES and GlutaMAX (Thermo Fisher Scientific) supplemented with 0.2 mg ml^−1^ ascorbic acid and 0.5 mg ml^−1^ albumin. Cells were sequentially treated with 4 μM CHIR99021 (Sigma-Aldrich) for 48 h, followed by 5 μM IWP2 (Sigma-Aldrich) for 48 h to induce a cardiac cell lineage. To drive differentiation toward an atrial cell phenotype, the cells were administered 1 μM retinoic acid at days 3–6. At day 6, simple CDM was added. Monolayers of beating iPSC-CMs were typically observed at days 7–8 onwards. The cells were maintained in RPMI 1640 + HEPES + GlutaMAX with the addition of 2% B27 supplement (Thermo Fisher Scientific).

For differentiation of cardiac fibroblasts, iPSCs were cultured in CDM with CHIR99021 for 1 d to induce a cardiac cell lineage as described above. After day 1, the medium was changed to CDM and cultured for an additional 24 h. After day 2, the medium was changed to a cardiac fibroblast-based medium (CFBM) comprising DMEM, 500 μg ml^−1^ albumin, 0.6 μM linoleic acid, 0.6 μg ml^−1^ lecithin, 50 μg ml^−1^ ascorbic acid, GlutaMAX, 1 μg ml^−1^ hydrocortisone hemisuccinate and 5 μg ml^−1^ insulin. CFBM was supplemented with 75 ng ml^−1^ bFGF, and media were replaced every other day until day 20 of differentiation.

### iPSC atrial cardiomyocyte calcium imaging assays

The atrial cardiomyocytes were differentiated on a 48-well plate setup (Corning). Only wells where there was spontaneous and consistent beating of the cardiomyocytes across the well were imaged. The rate of spontaneous beating was not controlled, as this would require pacing the monolayer of the sheets. However, the percentage change in the decay time was calculated for each well before and after co-culture with T_RM_ and non-T_RM_ cells. Given that this was a percentage change for each well, taking into account the same cardiomyocyte well density and beating characteristics, different wells could be compared by determining the percentage change in decay time for each well.

For T_RM_ cell purification, cells were initially stained with fixable Aqua Live/Dead cell stain, CD45 PE, CD45RO, CD8-AF700, CD3-APC/Cy7, CD69-PE, CD4-PE/Cy7 and PD1-PE-CF594 as previously discussed. Samples were then incubated at 4 °C for 20 min in the dark, after which samples were incubated for 15 min with MojoSort human anti-PE Nanobeads, followed by magnetic purification as per the manufacturer’s instructions (BioLegend) for CD45^+^ enrichement. Live T_RM_ cells were purified by FACS (LSRFortessa analyzer) and CD45RO^+^CD69^+^PD1^+^ cells. Non-T_RM_ cells were purified as CD45RO^+^CD69^−^PD1^−^ cells. Cells were added to cardiomyocytes at 2 × 10^3^ per well.

Calcium imaging was performed using the calcium indicator dye Fluo-4 AM (Invitrogen). Monolayers were illuminated with a single green light from a high-power LED with a center at wavelengths of 505 nm filtered with a bandpass excitation filter (490–510 nm). The LED power supply was custom built by Cairn Research. A Hamamatsu ORCA Flash 4.0 V2 camera was connected to a Nikon Eclipse TE200 inverted microscope, and a camera magnification objective of ×10 (numerical aperture 0.3) was used to provide a broad field of view. A bandpass emission filter (520–550 nm) was used to ensure that fluorescence emitted only after calcium indicator excitation was detected. HCImageLive (Hamamatsu) imaging software was used to acquire the data. The recording was then analyzed using custom in-house software (Queen Mary University of London). A total recording time of 20 s was used with a frame rate of 50 frames per second (image acquisition every 20 ms) to produce an image stack of 1,000 frames. Images were cropped to an area of 600 × 600 pixels that contained the projected image, and pixel binning was performed to improve signal-to-noise ratio. The recording was analyzed to drive a signal average calcium waveform from across the entire field of view. Relevant waveform statistics, including time to peak, time to 50% recovery to baseline (T_50_) and time to 90% recovery to baseline (T_90_), were determined. The data were exported as a CSV file for analysis in GraphPad Prism. Comparisons between different experimental conditions were, therefore, possible in a consistent and reproducible fashion, enabling assessment of how cardiomyocyte calcium flux changed before and after T_RM_ co-cultures.

### iPSC co-culture RNA expression analysis

Cells were washed with PBS, and RNA was extracted with an RNeasy Micro Kit (Qiagen) following the manufacturer’s instructions. Illumina sequencing was carried out at Novogene Bioinformatics Technology Co., Ltd. Raw FASTQ files were first trimmed using Trim Galore (version 0.6.7) and inspected for quality control using fastqc (version 0.11.9) and multiqc (version 1.12). Transcript and genome files were downloaded from GENCODE, release 43 (GRCh38.p13), to generate decoy index files with Salmon (version 1.10.1). Next, Salmon was used to perform transcript quantification with the additional parameter –gcbias. Salmon transcript quantifications were imported into R (version 4.1.0) to aggregate transcripts to genes using the package tximport (version 1.22.0). Genes with a read count of less than 5 were excluded from further analysis: rowSums(counts()) ≥ 5. Differential gene analysis was performed using DESeq2 (version 1.34.0). GSEA (version 4.3.2) was performed using normalized gene counts generated by DESeq2.

For RT–PCR, reverse transcription to cDNA was performed using High-Capacity RNA-to-cDNA kits (Applied Biosystems, Thermo Fisher Scientific). The relevant primer sequences can be found in Supplementary Table 15 (Thermo Fisher Scientific). Gene expression was performed using SYBR Green Supermix (Bio-Rad), as per the manufacturer’s instructions, and analyzed using the Light Cycler System (Roche). Relative gene expression values were determined using the ΔΔCT method and normalized to a stable reference housekeeping gene control (GAPDH). The control values were set at 1. Given that the ΔΔCT method is not normally distributed, the geometric mean was used for the representation of the data.

### Statistical analysis

Statistical significance was determined for continuous variables where two groups were assessed using two-tailed Student’s *t*-test where the data were parametric and Mann–Whitney *U*-test for non-parametric data. The χ^2^ test or Fisher’s exact test was used for categorical data. Data were analyzed using GraphPad Prism version 8. Normality was assessed using the Kolmogorov–Smirnov and Shapiro–Wilk tests. Where parametric data are represented, the mean and standard deviation values are reported, and, for non-parametric data, median and interquartile ranges are reported. A *P* value of less than 0.05 was considered statistically significant.

### Reporting summary

Further information on research design is available in the Nature Portfolio Reporting Summary linked to this article.

## Supplementary information

Reporting Summary

Supplementary Table 1 All patientsʼ clinical characteristics

Supplementary Table 2 Grouped patientsʼ clinical characteristics

Supplementary Table 3 Genes differentially expressed within each cluster

Supplementary Table 4 DEG between EAT versus SAT for each cluster

Supplementary Table 5 List of TCR clones

Supplementary Table 6 DEG of hyperexpanded clones in EAT versus AA

Supplementary Table 7 DEG between CD8^+^ T_RM_ clusters: cluster 2 (T_RM_-1), cluster 8 (T^RM^-2) and cluster 9 (T_RM_-3)

Supplementary Table 8 DEG between CD8^+^ T_RM_ clusters 2 versus 8

Supplementary Table 9 List of genes in the GeoMX platform

Supplementary Table 10 DEG deep in the tissue and border zone in the EAT

Supplementary Table 11 DEG deep in the tissue and border zone in the AA

Supplementary Table 12 DEG in the EAT border zone in AF versus SR patients

Supplementary Table 13 DEG in the AA border zone in AF versus SR patients

Supplementary Table 14 DEG of iPSC-CMs cultured with T_EM_ versus CD4^+^ T_RM_

Supplementary Table 15 List of primers for RT–PCR

## Source data

Source Data Fig. 3 Patients’ clinical characteristics for Fig. 3g–k.

Source Data Fig. 7 Statistical source data for Fig. 7b,f.

Source Data Extended Data Fig. 5 Statistical source data for Extended Data Fig. 5e,f.

## Data Availability

CITE-seq and RNA-seq raw and processed data are deposited in the Gene Expression Omnibus (GEO) under accession number GSE263154. Cell Ranger version 6.0.1 was used with default parameters to map all the data from the samples to the human reference genome (GRCh38; https://www.ncbi.nlm.nih.gov/datasets/genome/GCF_000001405.26/). Bulk TCR sequencing data are available at Zenodo (10.5281/zenodo.13318819) (ref. ^67^). The suite of tools for TCR sequencing analysis can be accessed at https://github.com/innate2adaptive/Decombinator. Spatial transcriptomic raw sequencing data have been deposited in the GEO under accession number GSE261363. Spatial profiling was carried out using the NanoString Technologies GeoMx Digital Spatial Profiler. iPSC-CM RNA-seq datasets have been deposited in the GEO under accession number GSE256520. Additional data generated in this study are provided in the Supplementary Information and Source Data sections.
