# Supplementary material for: Tissue-resident memory T cells in epicardial adipose tissue comprise transcriptionally distinct subsets that are modulated in atrial fibrillation
Source: Nat Cardiovasc Res. 2024 Aug 23;3(9):1067–82. doi: 10.1038/s44161-024-00532-x (PMC11399095; doi:10.1038/s44161-024-00532-x)
Supplement: Supplementary file 1 — Reporting Summary [file 44161_2024_532_MOESM1_ESM.pdf]

nature portfolio

Corresponding author(s): Paula Longhi

Last updated by author(s): 14/05/2024

Reporting Summary

Nature Portfolio wishes to improve the reproducibility of the work that we publish. This form provides structure for consistency and transparency in reporting. For further information on Nature Portfolio policies, see our Editorial Policies and the Editorial Policy Checklist.

Statistics

For all statistical analyses, confirm that the following items are present in the figure legend, table legend, main text, or Methods section.

n/a	Confirmed	
☐	☒	The exact sample size (*n*) for each experimental group/condition, given as a discrete number and unit of measurement
☐	☒	A statement on whether measurements were taken from distinct samples or whether the same sample was measured repeatedly
☐	☒	The statistical test(s) used AND whether they are one- or two-sided<br>*Only common tests should be described solely by name; describe more complex techniques in the Methods section.*
☐	☒	A description of all covariates tested
☐	☒	A description of any assumptions or corrections, such as tests of normality and adjustment for multiple comparisons
☐	☒	A full description of the statistical parameters including central tendency (e.g. means) or other basic estimates (e.g. regression coefficient) AND variation (e.g. standard deviation) or associated estimates of uncertainty (e.g. confidence intervals)
☒	☐	For null hypothesis testing, the test statistic (e.g. *F*, *t*, *r*) with confidence intervals, effect sizes, degrees of freedom and *P* value noted<br>*Give P values as exact values whenever suitable.*
☒	☐	For Bayesian analysis, information on the choice of priors and Markov chain Monte Carlo settings
☒	☐	For hierarchical and complex designs, identification of the appropriate level for tests and full reporting of outcomes
☒	☐	Estimates of effect sizes (e.g. Cohen's *d*, Pearson's *r*), indicating how they were calculated

Our web collection on statistics for biologists contains articles on many of the points above.

Software and code

Policy information about availability of computer code

Data collection	Chromium Single Cell 5' v2 sequencing was processed using the Cell Ranger (version 6.0.1). FASTQ files were generated using cellranger mkfastq (v.6.01) and gene expression reads were aligned to the human reference genome GRCh38 and counted using cellranger count (v.6.01.) VDJ reads were aligned to the GRCh38 VDJ reference dataset using cellranger vdj (v.6.01).<br>For RNAseq analysis, Raw FASTQ files were first trimmed using trim-galore (version 0.6.7) and inspected for quality control using fastqc (version 0.11.9) and multiqc (version 1.12). Transcript and genome files were downloaded from GENCODE, release 43 (GRCh38.p13) to generate a decoy index files with Salmon (version 1.10.1). Salmon transcript quantifications were imported into R (version 4.1.0) to aggregate transcripts to genes using the package tximport (version 1.22.0)<br>For Bulk TCR-seq analysis, TCR identification, error correction and CDR3 extraction was performed following a suite of tools available at github.com/innate2adaptive/Decombinator .

Data analysis	Expression matrices were analysed using the Seurat package257 (v4.0.03). DoubletFinder258 (v2.0.3) was employed for multiplet filtering. SCTransform259 (v0.3.2) was used for normalization and Seurat (v4.0.3) integration to remove batch effects. Analysis was performed using scRepertoire261 (v1.3.5) and STARTRAC262 (v0.1.0).<br>Differential gene analysis was performed using DESeq2 (version 1.34.0). GSEA (version 4.3.2) was performed using normalized gene counts generated by DESeq2.<br>Spatial transcriptomic data was analyzed using GeoMx® DSP Software v2.0. For spatial deconvolution, the_SpatialDecon_plugin.R (v1.2 Nanostring) was employed.<br>TCR frequency and similarity was analysed using the Immunarch package in R (v1.0.0).<br>Graphs were generated and analysed with GraphPad Prism version 8.<br>Flow cytometry data was analysed with FlowJo version 10.

For manuscripts utilizing custom algorithms or software that are central to the research but not yet described in published literature, software must be made available to editors and reviewers. We strongly encourage code deposition in a community repository (e.g. GitHub). See the Nature Portfolio guidelines for submitting code & software for further information.

Data

Policy information about availability of data

All manuscripts must include a data availability statement. This statement should provide the following information, where applicable:

- Accession codes, unique identifiers, or web links for publicly available datasets
- A description of any restrictions on data availability
- For clinical datasets or third party data, please ensure that the statement adheres to our policy

CITE-seq RNA sequencing raw and processed data are deposited in the Gene Expression Omnibus (GEO) under the accession number GSE263154. CellRanger v.6.0.1 was used with default parameters to map all the data from the samples to the human reference genome (GRCh38- https://www.ncbi.nlm.nih.gov/datasets/genome/GCF_000001405.26/). Bulk TCR-sequencing data are available at Zenodo https://doi.org/10.21203/rs.3.rs-3366081/v1. Suite of tools for TCR sequencing analysis can be access at github.com/innate2adaptive/Decombinator. Spatial Transcriptomic raw sequencing data have been deposited in GEO with the accession number GSE261363. Spatial profiling was carried out using the NanoString Technologies GeoMx® Digital Spatial Profiler. iPSC-cardiomyocytes RNA-seq datasets been submitted to GEO with the accession number GSE256520. Additional data generated in this study are provided in the Supplementary Information and Source Data sections.

Research involving human participants, their data, or biological material

Policy information about studies with human participants or human data. See also policy information about sex, gender (identity/presentation), and sexual orientation and race, ethnicity and racism.

Reporting on sex and gender	Information on sex has been collected and reported in the manuscript (clinical characteristics). Diabetes, hypertension, obesity, sleep-related breathing disorders, heart failure and ischaemic heart disease increase the risk of AF development/persistence with many of these risk factors being more prevalent in male, thus the higher proportion of males in our clinical cohort. For this reason, sex-based analysis was not possible due to low power.<br>Informed consent was obtained for all patients.
Reporting on race, ethnicity, or other socially relevant groupings	Although ethnicity information was obtained for most our patients, this was not reported in the study. Atrial Fibrillation (AF) is more prevalence among Caucasians compared with Blacks, and Asians and, therefore, 82% of our patients were white.
Population characteristics	all the covariant-relevant characteristics are indicated in the clinical characteristics (Supplementary Table 1) and were taking into consideration during propensity matching. APatients were matched for age, gender, BMI, diabetes, hypertension and procedure type (CABG/VR). Atrial fibrillation risk increases with age and metabolic syndrome (obesity, diabetes and metabolic syndrome). AF is common in participants with valvulopaties and affects more male than females.
Recruitment	Participants were selected based on inclusion and exclusion criteria. Inclusion criteria: Adult Adult (≥18 years) patients, undergoing on-pump open chest coronary artery bypass grafting (CABG) surgery, valve surgery or combined CABG/valve surgery were consented prior heart surgery at Barts Heart Centre, St Bartholomew's Hospital (London, UK) . Exclusion criteria included: congenital heart disease, underlying cardiomyopathies or ion channelopathies, primarily undergoing other cardiac surgical procedures (e.g. aortic surgery), off-pump CABG surgery, patients with active endocarditis, myocarditis or pericarditis, those with pre-existing inflammatory diseases (e.g. rheumatoid arthritis), active malignancy, patients on immunomodulatory or biologic drugs (e.g. tacrolimus, anti-TNF-α agents), peri-operative rhythm control therapies e.g. use of amiodarone, post-operative haemodynamic shock, uncorrected potassium derangement (K<3.3 or K>5.8) or uncorrected magnesium derangement (Mg<0.5 or Mg>1.5) detected on laboratory blood sample analysis. No other selection criteria were employed .<br>Participants were consented prior surgery. The Barts Heat Centre is one of the largest cardiac unit in Europe serving an ethnically diverse community and therefore not all of them speak and/or read English. Experienced translators and English speaking family members were present at the time of consent to limit sampling biases. However, despite the diverse population, this is a single-centre study which could bias towards a population with specific clinical characteristics.
Ethics oversight	East of England - Cambridge Central Research Ethics Committee (REC). Participants were screened and gave informed written consent for study participation as per local research procedures and Good Clinical Practice guidance through the Barts BioResource (Research Ethics Committee reference: 14/EE/0007).

Note that full information on the approval of the study protocol must also be provided in the manuscript.

Field-specific reporting

Please select the one below that is the best fit for your research. If you are not sure, read the appropriate sections before making your selection.

☒ Life sciences ☐ Behavioural & social sciences ☐ Ecological, evolutionary & environmental sciences

For a reference copy of the document with all sections, see nature.com/documents/nr-reporting-summary-flat.pdf

Life sciences study design

All studies must disclose on these points even when the disclosure is negative.

Sample size	Power calculations were based on the percentage of Trm cells, with CD4+TRM cells giving the higher effector size. Power calculations were calculated using T-test, with a mean of 37.8 and 56.5 and SD of 20.3, and to achieve a power of 0.8 with a significant level of 0.05. For in vitro Trm cultures, power calculations were based in published data in Bourdely P et al. doi: 10.1016/j.immuni.2020.06.002, requiring n=5 donors/condition (Effect size 2.96, alpha error 0.05 and 95% power)
Data exclusions	Participant on sinus rhythm (SR) prior surgery were monitored during hospital stay for the development of Post-operative AF (POAF), defined as an ECG of >30seconds of AF during the 7-day post-operative period. Those that developed POAF were excluded post data acquisition from the study as they could not be classified as SR any longer.
Replication	As this is a clinical observational study, with limited human sample collection, reproducibility of our findings were based on the use of different technical approaches to confirm the initial findings obtained with flow cytometry. Data are based on biological replicates with flow cytometry performed on 153 participants (72 after propensity matching). In vitro co-cultured assays were performed with 5 biological replicates in three experimental repeats. Bulk TCR-seq and RAN-sea analysis were performed in 5 biological repeats.
Randomization	No randomization was applied as was not applicable to the study. Covariants were controlled with propensity matching.
Blinding	Patients samples were labeled with nonidentifying numbers and sample analysis was performed blindly by two investigators.

Reporting for specific materials, systems and methods

We require information from authors about some types of materials, experimental systems and methods used in many studies. Here, indicate whether each material, system or method listed is relevant to your study. If you are not sure if a list item applies to your research, read the appropriate section before selecting a response.

Materials & experimental systems

n/a	Involved in the study	
☐	☒	Antibodies
☐	☒	Eukaryotic cell lines
☒	☐	Palaeontology and archaeology
☒	☐	Animals and other organisms
☒	☐	Clinical data
☒	☐	Dual use research of concern
☒	☐	Plants

Methods

n/a	Involved in the study	
☒	☐	ChIP-seq
☐	☒	Flow cytometry
☒	☐	MRI-based neuroimaging

Antibodies

Antibodies used

TotalSeq antibodies were purchased from Biolegend.
TotalSeq™-C0138 anti-human CD5 (1:200); clone: UCHT2; CAT: 300637; Lot: B309305
TotalSeq™-C0358 anti-human CD163 (1:200); clone: GHI/61; Cat: 333637; Lot: B322549
TotalSeq™-C0160 anti-human CD1c (1:200); Clone: L161; Cat: 331547; Lot: B306006
TotalSeq™-C0049 anti-human CD3 (1:200); Clone: SK7; Cat: 344849; Lot: B306005
TotalSeq™-C0072 anti-human CD4 (1:200); Clone: RPA-T4; Cat: 300567; Lot: B310155
TotalSeq™-C0080 anti-human CD8a (1:200); Clone: RPA-T8: Cat: 301071; Lot: B315106
TotalSeq™-C0087 anti-human CD45RO (1:100); Clone: UCHL1; Cat: 304259; Lot: B335899
TotalSeq™-C0148 anti-human CD197 (1:100),Clone: G043H7; Cat: 353251; Lot: B330975
TotalSeq™-C0146 anti-human CD69 (1:100); Clone: FN50; Cat: 310951; Lot: B337757
TotalSeq™-C0088 anti-human CD279 (1:100); Clone: EH12.2H7; Cat: 329963; Lot: B313421
TotalSeq™-C1046 anti-human CD88 (1:200); Clone: S5/1; Cat: 344319; Lot: B306415
Addition antibodies
CD197-FITC (Biolegend, Cat: 353216,1:200 dilution, clone 353216), CD19-PerCP-Cy5.5 (Biolegend, Cat 302228, Clone H1B19, 1:200 dilution), CD45RO-BV421 (Biolegend, Cat 304224,Clone UCHL1, 1:200 dilution), CD335-BV605 (Biolegend, Cat 331926, Clone 9E2, 1:200 dilution), CD45-BV785 (Biolegend, Cat 304048, Clone HI30, Lot B284678, 1:200 dilution), CD127-APC (Biolegend, Cat 351342, Clone A019D5, Lot B280742 1:200 dilution), CD8-AF700 (Biolegend, Cat 300920, CloneHIT8a, 1:500 dilution), CD3-APC/Cy7 (Biolegend, Cat 300318, Clone HIT3a, 1:200 dilution), CD69-PE (Biolegend, Cat 310906,Clone FN50, 1:200 dilution), CD4-PE/Cy7

(Biolegend, cat 357410, Clone A161A1, 1:200 dilution), PD1-PE-CF594 (Biolegend, cat 329940, Clone EH12.2H7, 1:200 dilution), KLRG1-SB702 (eBioscience, Cat 15824202, Clone 13F12F2 1:200 dilution), CD303-FITC (Biolegend, Cat 354208, Clone 201A, 1:200 dilution), CD123-PerCP/Cy5.5 (Biolegend, Cat 306016, Clone 6H6, 1:200 dilution), CD206-BV421 (Biolegend, Cat 321126, Clone 15-2 1:200 dilution), CD3-BV605 (Biolegend, Cat 317322, Clone OKT3, 1:200 dilution), CD19-BV605 (Biolegend, Cat 302244, Clone HIB19, 1:200 dilution), CD14-APC (Biolegend, Cat 325608, Clone HCD14, 1:200 dilution), CD16-AF700 (Biolegend, Cat 302026, Clone 3G8, 1:500 dilution), CD1c-APC/Cy7 (Biolegend, Cat 331520, Clone L1G1, 1:200 dilution), Clec9A-PE (Biolegend, Cat 353804, Clone 8F9, 1:200 dilution), CD1a-PE-CF594 (Biolegend, Cat 300132, Clone H149, 1:200 dilution), and CD141-PE/Cy7 (Biolegend, Cat 344110,Clone M80, 1:200 dilution), IFNg-APC (Biolegend, Cat 502511, Clone 4S.B3, 1:200 dilution), IL17-APC/Cy7 (Biolegend, Cat 512319, Clone BL168, 1:200 dilution), IL22-PE (Biolegend, Cat 366703, Clone 2G12A41, 1:200 dilution).

Validation	Antibodies were previously validated in Vyas et al. (PMID: 34283808). Total-seq antibodies were validated by flow cytometry using the same fluorescent-labeled antibody clones.

Eukaryotic cell lines

Policy information about cell lines and Sex and Gender in Research

Cell line source(s)	HipSci deposited by the Wellcome Trust Sanger institute into the Culture Collections archive (UK Health Security Agency, UK).
Authentication	Atrial phenotype upon differentiation was validated by gene expression profile and electrical changes.
Mycoplasma contamination	negative
Commonly misidentified lines (See ICLAC register)	No Misidentified Cell Lines were used in the study

Flow Cytometry

Plots

Confirm that:

☒ The axis labels state the marker and fluorochrome used (e.g. CD4-FITC).

☒ The axis scales are clearly visible. Include numbers along axes only for bottom left plot of group (a 'group' is an analysis of identical markers).

☒ All plots are contour plots with outliers or pseudocolor plots.

☒ A numerical value for number of cells or percentage (with statistics) is provided.

Methodology

Sample preparation	AA samples were enzymatically digested with 675U collagenase I (Sigma-Aldrich), 187.5U collagenase XI (Sigma-Aldrich) and 10U DNase (Sigma-Aldrich) in 1ml of Hanks' buffered salt solution at 37°C with 225rpm agitation for 45 mins. Adipose tissue samples were digested with Collagenase II and DNase at 37°C with 225rpm agitation for 30 mins. PBMCs were isolated using Ficoll-Paque PLUS (Cytiva) as per manufacturer's instructions. Single cell suspensions were obtained following centrifugation and red cell lysing prior to antibody staining
Instrument	Data was acquired on a Cytoflex (Beckman Coulter)
Software	FlowJo version 10 software.
Cell population abundance	TRM cells comprise a 40 to 80% of CD4+ or CD8+ T cell respectively and proximately a 20-30% of immune cells. Post-sort purity was assessed by sorted cell analysis by LSR Fortessa analyzer.
Gating strategy	Gating strategy is defined in the supplementary data.

☒ Tick this box to confirm that a figure exemplifying the gating strategy is provided in the Supplementary Information.
