# Supplementary material for: Tissue-resident memory T cells in epicardial adipose tissue comprise transcriptionally distinct subsets that are modulated in atrial fibrillation
Source: Nat Cardiovasc Res. 2024 Aug 23;3(9):1067–82. doi: 10.1038/s44161-024-00532-x (PMC11399095; doi:10.1038/s44161-024-00532-x)
Supplement: Supplementary file 2 — All patientsʼ clinical characteristics [file 44161_2024_532_MOESM2_ESM.pdf]

Supplementary Table 1. Patients' clinical characteristics (n=153)

Variable	All patients (153)
**Age (Years)**	66.1 ± 10.1
**Male Gender (%)**	115 (75)
**Body Mass Index (kg/m^2)**	28.2 ± 4.5
**Diabetes (%)**	41 (27)
**Hypertension (%)**	110 (71)
**Smoking History (%)**	8 (44)
**Prior Myocardial Infarction (%)**	48 (31)
**Left Ventricular Ejection Fraction (%)**	58 (55-62)
**Pre-operative use of beta blockers (%)**	95 (62)
**Pre-operative use of statins (%)**	114 (74)
**Pre-operative C-reactive protein (mg/L)**	1 (0-4)
**Pre-operative neutrophil:lymphocyte**	2.1 (1.6-3.0)
**Indexed left atrial size (cm/m^2)**	1.9 (1.9-2.5)
**Coronary artery bypass surgery (%)**	77 (50)
**Valve surgery (%)**	59 (38)
**Combination coronary artery bypass/Valve surgery (%)**	18 (12)
