# Supplementary material for: Tissue-resident memory T cells in epicardial adipose tissue comprise transcriptionally distinct subsets that are modulated in atrial fibrillation
Source: Nat Cardiovasc Res. 2024 Aug 23;3(9):1067–82. doi: 10.1038/s44161-024-00532-x (PMC11399095; doi:10.1038/s44161-024-00532-x)
Supplement: Supplementary file 3 — Grouped patientsʼ clinical characteristics [file 44161_2024_532_MOESM3_ESM.pdf]

Supplementary Table 2. Summary of baseline patient characteristics.

VARIABLE	PRE-EXISTING AF (N=38)	SINUS RHYTHM (N=84)	POAF (N=31)	*P* VALUE
**Age (Years)**	69.9 ± 7.9	62.3 ± 10.5	71.8 ± 7.0	<0.0001
**Male Gender (%)**	28 (74)	43 (71)	20 (84)	0.39
**Body Mass Index (kg/m^2)**	25.4.4 (23.5-29.8)	29.3 (24.6-31.5)	29.8 (25.9-31.9)	0.03
**Diabetes (%)**	10 (26)	24 (29)	7 (23)	0.81
**Hypertension (%)**	23 (61)	63 (75)	24 (77)	0.19
**Prior Myocardial Infarction (%)**	8 (21)	30 (36)	10 (32)	0.27
**Left Ventricular Ejection Fraction (%)**	60 (53-63)	58 (52-60)	59 (55-62)	0.48
**Pre-operative use of beta blockers (%)**	25 (66)	52 (62)	18 (58)	0.80
**Pre-operative use of statins (%)**	26 (68)	64 (76)	24 (77)	0.60
**Pre-operative C-reactive protein (mg/L)**	1 (0-3)	1 (0-3)	0 (0-5)	0.92
**Pre-operative white cell count (10^9 g/L**	7.0 (5.9-8.4)	8.0 (6.7-10.0)	7.3 (6.9-8.7)	0.05
**Indexed left atrial size (cm/m^2)**	2.5 (2.0-3.0)	1.9 (1.9-2.1)	2.0 (1.9-2.4)	<0.0001
**Post-operative intercurrent illness (%)**	2 (5)	10 (12)	4 (13)	0.48
**Coronary artery bypass surgery (%)**	9 (24)	55 (65)	13 (42)	<0.0001
**Valve surgery (%)**	26 (68)	22 (26)	10 (32)	<0.0001
**Combination coronary artery bypass/Valve surgery (%)**	3 (9)	7 (8)	8 (26)	0.03

The Welch's one-way ANOVA test with multiple comparisons was used for continuous parametric variables, Kruskall-Wallis test for non-parametric continuous data and Chi squared test/Fisher's exact test for categorical variables. The pre-existing AF and POAF groups were compared against SR. Post-operative intercurrent illness here includes pneumonia, wound infection, development of stroke or need for repeat sternotomy (e.g. for significant pericardial effusion drainage).
