# Supplementary material for: Tissue-resident memory T cells in epicardial adipose tissue comprise transcriptionally distinct subsets that are modulated in atrial fibrillation
Source: Nat Cardiovasc Res. 2024 Aug 23;3(9):1067–82. doi: 10.1038/s44161-024-00532-x (PMC11399095; doi:10.1038/s44161-024-00532-x)
Supplement: Supplementary file 16 — List of primers for RT–PCR [file 44161_2024_532_MOESM16_ESM.pdf]

Supplementary Table 15. List of primers for RT-PCR.

Primer	Forward sequence (5’ to 3’)	Reverse sequence (5’ to 3’)
GAPDH	GGCTCATGACCACAGTCCA	CACATTGGGGGTAGGAACAC
NPPA	ACAGGATTGGAGCCCAGAG	GGAGCCTCTTGCAGTCTGTC
TBX18	ACTGTCTTCACAACCGTCAC	CTTCCAAACCCATTCTGTTGC
KCNN3	CCTGTATGAGTCAGCCTTTC	AGCTCTAGGGACTTCTAACC
KCNQ2	TCTCCTGCCTCGTGCTGTCT	GCGTAGGTGTCAAAGTGGTC
SCN2A	TTCATTGGATGGGAATGGTACT	CTGTTGCCACAAAGCAGAGC
DDX58	AGAGCACTTGTGGACGCT TT	AGCAACTGAGGTGGCAATCA
COL3A1	TCTGCCATCCTGAACTCAAGA	TGCATGTTTCCCCAGTTTCC
COL1A1	GATTCCCTGGACCTAAAGGTGC	AGCCTCTCCATCTTTGCCAGCA
COL6A1	ACAGTGACGAGGTGGAGATCA	GATAGCGCAGTCGGTGTAGG
MMP1	CTGAAGGTGATGAAGCAGCC	AGTCCAAGAGAATGGCCGAG
DDR2	CTTTGGCTGGACTCTCCTGGCTC	TCCCATGACGGTTCCGCCAAGA
